# Supplementary material for: Clinical use of an emergency manual by resuscitation teams and impact on performance in the emergency department: a prospective mixed-methods study protocol
Source: BMJ Open. 2023 Oct 17;13(10):e071545. doi: 10.1136/bmjopen-2022-071545 (PMC10583077; doi:10.1136/bmjopen-2022-071545)
Supplement: Supplementary data [file bmjopen-2022-071545supp001.pdf]

Appendix A: Structured Interview Guide for Reported Specific Cases

Introduction: You have requested to speak with us about the use of the Emergency Manual during the management of a specific patient in the resuscitation room. We are interested in understanding how the Emergency Manual is used, how it impacts on actual clinical care and how it can be improved.

Important information:
This project has been approved by the Swedish Ethical Review Authority
The names of specific health care personnel are not recorded.
This information obtained through this interview will not be used for any institutional process for assessing clinical management.

Background Information

Date of the interview:	
Date specific case managed:	
Interviewee's profession:	☐ Nursing assistant ☐ Nurse ☐ Resident ☐ Specialist. Physician's specialty:
Physician in charge of case	☐ Resident ☐ Specialist. Specialty:
Patient age and sex	____________ years. ☐ Male ☐ Female
Presenting complaint	
Suspected diagnosis	

Events or concerns relating to EM use (free text summary, professions of individuals recorded but names NOT recorded)

Emergency Manual Use

Sections of EM relevant to the event	
Mode of EM use	☐ Do-Confirm ☐ Read-Do ☐ Sampling

Assessed impact of EM use on patient care (clinical decisions regarding diagnosis, treatment; free text summary)

Summary of Impact of EM use on patient care: ☐ Negative ☐ Positive

Assessed impact of EM use on team members/teamwork (organization, communication; free text summary)

Summary of Impact of EM use on team members/teamwork: ☐ Negative ☐ Positive

Verbatim summary of how the impact of EM access on the specific case:

Suggestions for improvement (free text summary):

