# Supplementary material for: Clinical use of an emergency manual by resuscitation teams and impact on performance in the emergency department: a prospective mixed-methods study protocol
Source: BMJ Open. 2023 Oct 17;13(10):e071545. doi: 10.1136/bmjopen-2022-071545 (PMC10583077; doi:10.1136/bmjopen-2022-071545)
Supplement: Author's manuscript [file bmjopen-2022-071545.draft_revisions.pdf]

BMJ Open

BMJ Open is committed to open peer review. As part of this commitment we make the peer review history of every article we publish publicly available.

When an article is published we post the peer reviewers' comments and the authors' responses online. We also post the versions of the paper that were used during peer review. These are the versions that the peer review comments apply to.

The versions of the paper that follow are the versions that were submitted during the peer review process. They are not the versions of record or the final published versions. They should not be cited or distributed as the published version of this manuscript.

BMJ Open is an open access journal and the full, final, typeset and author-corrected version of record of the manuscript is available on our site with no access controls, subscription charges or pay-per-view fees (http://bmjopen.bmj.com).

If you have any questions on BMJ Open's open peer review process please email info.bmjopen@bmj.com

BMJ Open

Clinical use of an emergency manual by resuscitation teams and impact on performance in the emergency department—a prospective mixed-methods study protocol

Journal:	*BMJ Open*
Manuscript ID	bmjopen-2022-071545
Article Type:	Protocol
Date Submitted by the Author:	04-Jan-2023
Complete List of Authors:	Dryver, Eric; Skåne University Hospital Lund, Department of Emergency and Internal Medicine; Lund University, Department of Clinical Sciences at Lund<br>Olsson de Capretz, Pontus; Skanes universitetssjukhus Lund, Department of Emergency and Internal Medicine; Lund University, Department of Clinical Sciences at Lund<br>Mohammad, Mohammed; Skåne University Hospital Lund, Department of Emergency and Internal Medicine<br>Armelin, Malin; Skåne University Hospital Lund, Department of Emergency and Internal Medicine<br>Dupont, William D. ; Vanderbilt University Medical Center, Department of Biostatistics<br>Bergenfelz, Anders; Lund University, Department of Clinical Sciences at Lund<br>Ekelund, Ulf; Skåne University Hospital Lund, Department of Emergency and Internal Medicine; Lund University, Department of Clinical Sciences at Lund
Keywords:	ACCIDENT & EMERGENCY MEDICINE, INTENSIVE & CRITICAL CARE, Protocols & guidelines < HEALTH SERVICES ADMINISTRATION & MANAGEMENT

SCHOLARONE™
Manuscripts

TITLE PAGE

Clinical use of an emergency manual by resuscitation teams and impact on performance in the emergency department—a prospective mixed-methods study protocol

AUTHORS

Eric Dryver[*†‡], M.D. eric.dryver@med.lu.se

Pontus Olsson de Capretz[*†], M.D. pontus.olsson_de_capretz@med.lu.se

Mohammed Mohammad[*], M.D. mohammed.mohammad@skane.se

Malin Armelin[*], RN, MSN Malin.Armelin@skane.se

William D Dupont[§], Ph.D. william.dupont@vumc.org

Anders Bergenfelz[†], M.D., Ph.D. anders.bergenfelz@med.lu.se

Ulf Ekelund[*†], M.D., Ph.D. ulf.ekelund@med.lu.se

* Department of Emergency and Internal Medicine, Skåne University Hospital at Lund, Sweden

† Department of Clinical Sciences at Lund, Lund University, Sweden

‡ Practicum Clinical Skills Centre, Lund, Sweden

§ Department of Biostatistics, Vanderbilt University Medical Center, USA

CORRESPONDING AUTHOR

Eric Dryver

Department of Emergency Medicine

Skåne University Hospital at Lund

Klinikgatan 15

22185 Lund

Sweden

eric.dryver@med.lu.se

WORD COUNT

3980

For peer review only

ABSTRACT

Introduction

Simulation-based studies indicate that crisis checklist use improves management of patients with critical conditions in the emergency department (ED). An interview-based study suggests that use of an emergency manual (EM)—a collection of crisis checklists—improves management of perioperative crises. To date, there are no prospective studies of EMs during clinical practice.

Methods and analysis

This six-month-long study prospectively evaluates a digital EM during management of priority 1 patients in the Skåne University Hospital at Lund's ED. Resuscitation teams are encouraged to use the EM after a management plan has been derived ("Do-Confirm"). The documentation nurse is assigned the role of activating and reading from the EM, and checklists are displayed on a large screen visible to all team-members. Whether the EM is activated, and which sections are displayed, are automatically recorded. Interventions performed thanks to Do-Confirm EM use are registered by the nurse. Fifty cases featuring such interventions are reviewed by specialists in emergency medicine blinded to whether the interventions were performed prior to or after EM use. All interventions are graded as indicated, of neutral relevance or not indicated. The primary outcome measures are the proportions of interventions performed thanks to Do-Confirm EM use graded as indicated, of neutral relevance, and not indicated. A secondary outcome measure is the team's subjective evaluation of the EM's value on a Likert scale of 1 to 6. Team members are encouraged to report events related to EM use, and information from these events are extracted through structured interviews.

For peer review only - http://bmjopen.bmj.com/site/about/guidelines.xhtml

Ethics and dissemination

The study has been approved by the Swedish Ethical Review Authority (Dnr 2022-01896-01). Results will be published in a peer-reviewed journal and abstracts submitted to national and international conferences to disseminate our findings.

Registration

The trial is registered at clinicaltrials.gov (NCT 05649891).

For peer review only

For peer review only - http://bmjopen.bmj.com/site/about/guidelines.xhtml

STRENGTHS AND LIMITATIONS OF THIS STUDY

- To date, studies of Emergency Manuals (EM)—collections of crisis checklists— have been simulation- or interview-based. This study prospectively evaluates an EM during the management of unselected, consecutive priority 1 patients in an emergency department over a six-month period.
- Some of the data on patient characteristics and EM use are automatically registered, while other EM use data are actively registered by the documentation nurse.
- The study aims to determine whether interventions carried out thanks to checklist use are indicated, of neutral relevance or not indicated based on the clinical context, as assessed by external reviewers.
- The external review process is based on information available from patient records, which may not convey all the relevant factors affecting clinical decisions.
- The study is not a randomized-controlled trial and is not powered nor designed to detect causal relationship between EM use and patient outcome.

For peer review only

INTRODUCTION

Checklists

A checklist is a cognitive aid—consisting of a list of assessments or actions—designed to be carried out systematically. The function of checklists is to improve task performance; checklists are essentially knowledge-translation tools that promote completeness, consistency and alignment with best practice guidelines when carrying out a task [1]. Checklists work by directing focus and providing facts [2]. Checklists are also believed to improve performance through other mechanisms, such as reducing mental workload, promoting a shared mental model among team-members and empowering team-members to voice concerns [3-5].

There are two basic modes of checklist use [6]. In Read-Do mode, checklists are used as the task is performed. During the Do-Confirm mode, checklists are used after task performance to verify completeness. Cognitive aids may even be used in a non-systematic manner to retrieve specific information (for instance the dosage of a specific medication in a specific setting), so-called Sampling mode [6, 7]. Checklists may be used by one or several people, silently or read aloud [6], and can be categorized according to the circumstances in which they are used: normal (or routine) situations, emergency situations, and atypical (or non-normal) situations [6, 8].

Checklists for routine situations in health care

The World Health Organization (WHO) Surgical Safety Checklist [9] and the Surgical Safety Patient System [10] are examples of checklists used to improve performance during routine surgical procedures. There is substantial, robust, international evidence that use of the WHO Surgical Safety Checklist reduces mortality and complications associated with general surgery [11]. A checklist designed to minimize bloodstream infections associated with the

For peer review only - http://bmjopen.bmj.com/site/about/guidelines.xhtml

routine insertion of central venous catheters in the intensive care unit is another example of a checklist associated with improved performance during routine health care delivery [12-14].

Checklists for health care emergencies

Stress affects attention and information retrieval from memory [15, 16], and hence checklists may play a particular role in improving performance during stressful emergencies [1, 4]. Due to the unexpected, low-frequency nature of medical emergencies, crisis checklists have mainly been studied using simulation. One study reported a reduction in the frequency of unperformed lifesaving processes of care from 23% to 6% associated with checklist access during simulated surgical crises [17]. Another study reported that checklist use increased the number of key items performed during scenarios in a simulated intensive care unit from 7 to 9 [18].

Two studies evaluated crisis checklists in a simulated resuscitation room. One study evaluated the use of an Emergency Protocols Handbook through four scenarios and reported that checklist use decreased error rate from 39% to 19% [19]. The other study evaluated a collection of four checklists through four scenarios; checklist use was associated with an increased frequency of hemodynamic stabilization and diagnosis recognition, as well as an increased adherence to critical steps (56% without checklist, 82% with checklists) [20].

A simulation-based, randomized controlled trial of medical crisis checklists was performed in four resuscitation rooms using actual resuscitation teams during their clinical shifts [21]. Eight medical crises were simulated. With checklist access, the median percentage of key interventions performed was 86%; without access, the median percentage was 39%.

For peer review only - http://bmjopen.bmj.com/site/about/guidelines.xhtml

Evidence that collections of crisis checklists—Emergency Manuals (EM)—improve actual clinical care is limited. Several reports document successful management of perioperative crises thanks to the use of operating room EMs [22-25]. An interview-based study of anaesthesiologists involved in perioperative crises in 69 patients revealed that EM use led to identifying errors of omission in 59% of cases, while lack of EM use was associated with self-identified errors of omission or delays in intervention performance in 56% of cases [5].

Systematic crisis checklist evaluation in the clinical setting

Performance is the end-product of complex interactions between people, the tools at their disposal, and the physical and cultural environments they work in [26]. Checklists do not work in isolation, and their impact on performance is contingent not only upon their content and design but also on when, where, how and by whom they are used. Simulation-based trials and interview-based studies [27] provide limited evidence for how checklists may actually impact resuscitation team performance in a specific clinical setting. Several authors have called for pilot-studies that systematically evaluate crisis checklists in the clinical setting [5, 19, 21, 27].

Experts emphasize the importance of establishing the rational for EM use, fostering a sense of local EM ownership, ensuring access and familiarity with the tool, and customization [28]. In particular, an explicit protocol is likely required to ensure that checklists are used as intended. A simulation-based study reported that the mere access to crisis checklists did not ensure their systematic use; only when an assigned team-member read aloud the steps on the checklist were all critical actions performed [29]. The Surgical Safety Checklist Implementation Manual emphasizes the importance of clearly assigning the role of checklist

For peer review only - http://bmjopen.bmj.com/site/about/guidelines.xhtml

implementation to a specific person[30] and an interview-based study of EM use during perioperative crises reported beneficial effects of a reader-leader combination [5].

Challenges to implementation and evaluation of an EM in the ED include the following:

- the number of possible presenting complaints, acute conditions and pre-existing co-morbidities among priority 1 patients is large, and the degree of actual acuity is variable
- the number of personnel that work in the resuscitation room is large, and each personnel has variable context-specific degrees of expertise; managing a patient with acute pulmonary edema may be "routine" for a seasoned physician and an "emergency" for a junior resident
- Do-Confirm mode of checklist use is best suited to quantify the added value of checklist use, while Read-Do and Sampling modes may be best suited for checklist use during an actual crisis

These challenges have informed the study design.

Objectives

The primary objective of the study is to determine whether Do-Confirm EM use leads to the performance, by resuscitation teams managing priority 1 patients in the ED, of additional interventions that are deemed indicated, of neutral relevance or not indicated given the clinical context. Secondary study objectives are

- determining the frequency and modality (Do-Confirm, Read-Do, Sampling) with which the EM is used and whether these changed significantly over the study period
- determining whether Do-Confirm EM use in selected cases where other modes were used might have led to the performance of additional indicated interventions

For peer review only - http://bmjopen.bmj.com/site/about/guidelines.xhtml

- determining the degree to which resuscitation teams find the EM helpful, and under which circumstances

METHODS AND ANALYSIS

Setting, patient population and personnel

The study will be carried out in the resuscitation room of the ED of Skåne's University Hospital at Lund (referred to hereafter as Lund's ER). Lund’s ER is designed for the simultaneous management of three priority 1 patients. EM access during clinical practice will be provided in September 2023. The study population consists of consecutive priority 1 patients managed in the Lund's ER during the six-month period prior to, and the six-month pilot study period following EM implementation. Patients are deemed to be priority 1 based on vital signs and symptoms according to the Rapid Emergency Triage and Treatment System (http://predicare.se/) the most commonly used triage system in Sweden. The yearly number of priority 1 patients treated in Lund's ER is roughly 4000 and consists of adult patients (> 18 years) with medical or surgical emergencies and patients of all ages with level 1 trauma. Most of these patients are brought to the ER via ambulance, and only occasionally are they identified at the triage or at later stage of ED care. All priority 1 patients are managed immediately in the ER by multidisciplinary resuscitation teams. The core resuscitation team consists of a resident or specialist in emergency medicine, two nurses and one nursing assistant. When the patient is especially critical (for instance cardiac arrest, level 1 trauma, upper airway obstruction), additional personnel (anaesthesiologists, surgeons, cardiologists, otorhinolaryngologists) supplement the resuscitation team.

For peer review only - http://bmjopen.bmj.com/site/about/guidelines.xhtml

Emergency Manual: content and format

The Emergency Manual consists of diagnosis checklists, problem checklists, procedure checklists and fact sheets in digital form. The checklists and fact sheets are accessed through a tablet computer located at each of the three resuscitation sections in the ER and displayed on a large screen visible to all members of the resuscitation team. Diagnosis checklists consist of lists of interventions to consider during the management of a suspected diagnosis. Each intervention is associated with a popover window displaying indications, contraindications or risks associated with the intervention, and details regarding how the intervention is performed, as described in a previous study [21]. Problem checklists consist of lists of interventions and diagnoses to consider based on the patient's presenting symptoms. The popover windows display key clinical features suggestive of specific diagnoses. Procedure checklists provide step-by-step guidance for the performance of key procedures as well as pictures. Fact sheets provide information such as medical dosages for sedation and anaesthesia for patients of all ages.

The collection of checklists and fact sheets covers the majority of symptoms, diagnoses and situations that may present in the ER, in accordance with the current European Core Curriculum in Emergency Medicine [31]. The format and design of each checklist or fact sheet is informed by the literature pertaining to emergency and abnormal checklists in the aviation industry [32-34], articles on medical checklists [6, 35-37] and a selection of EMs [38-41]. The content is customized to local circumstances in Lund's ED (available medications, medication locations, local routines and phone numbers). EM content and format are further developed through iterative, rapid-cycle simulation-based testing and review by specialists, residents, nurses and nursing assistants who work clinically in Lund's ER. Internet access to the EM is provided to the ED personnel prior to and during the study to

For peer review only - http://bmjopen.bmj.com/site/about/guidelines.xhtml

allow for familiarization and suggestions for improvement. Each checklist and fact sheet is approved by at least one specialist in emergency medicine and one nurse, both of whom work clinically in Lund's ER.

Emergency Manual: protocol for use

The documentation nurse during priority 1 patient management is assigned the specific task of activating the digital EM. The default mode of EM use is Do-Confirm. The team first manages priority 1 patients without support from the EM. The documenting nurse then displays the checklist(s) relevant to the specific circumstances and reads aloud each potential intervention/diagnosis for team consideration. The documenting team-member registers on the digital checklist whether each intervention is:

- deemed indicated and has already been carried out
- deemed irrelevant or contraindicated
- deemed indicated and will be carried out thanks to EM use

The physician responsible for patient management has the mandate to request that the EM not be used, to pause or terminate EM use at any time, and to depart from the recommendations featuring on the checklist. Physicians may also request that the EM be displayed directly and used in Read-Do mode or for retrieval of specific information (Sampling mode). After EM use, teams grade the usefulness of the EM on a 1-6 Likert scale prior to logging out. Teams can also electronically request contact with the investigators to report specific events related to EM use.

Educational campaign

An educational campaign targeting all personnel who work in Lund's ER precedes the implementation of the EM in clinical practice. Evidence for the potential benefits of an EM—

For peer review only - http://bmjopen.bmj.com/site/about/guidelines.xhtml

including results from a locally-performed crisis checklist study [21]—is presented, as well as the protocol for EM use. Documentation nurses during priority 1 patient management are taught to log in to the EM, display and navigate between checklists, open popover windows, document whether interventions were not indicated, done before or thanks to checklist access, record the team's opinion of the EM's value, request contact with the investigators, and log out.

Data acquisition

The following data pertaining to each priority 1 patient managed during the six-month period prior to, and the six-month pilot study period following implementation of the EM in clinical practice, are extracted from Region Skåne's clinical databases and the national data registry SVAR (Svenska Akutvårdsregistret):

- patient age and sex, presenting complaint and vital signs
- presumptive diagnosis in the ER
- time spent in the ER, in the ED, and admitted to the hospital
- whether the patient was admitted to the intensive care unit, and if so duration of admission
- seven-day and thirty-day mortality

The following data regarding EM use are recorded on a dedicated computer located within the hospital's firewall each time the EM is activated during the six-month pilot study period:

- log-in and log-out times
- identification number of the nurse who logged in, level of the most senior physician who was physically present at the bedside during patient management, and patient identification number (which is immediately encrypted)
- checklists and fact sheets that were displayed

For peer review only - http://bmjopen.bmj.com/site/about/guidelines.xhtml

- whether interventions were performed before or thanks to checklist access when the EM was used in Do-Confirm mode, or whether the EM was used in Read-Do or Sampling mode instead
- the team's subjective evaluation of the value of the EM according to a 1-6 Likert scale
- whether prompt contact with the research team is requested

The following data is extracted from the medical records of 150 selected cases (see below):

- baseline information regarding the patient's age, sex, known medical background at the time of presentation and current medications
- clinical information regarding the patient's presentation to the ER: ambulance report, history that can be obtained in the ER, vital signs, physical findings, electrocardiogram, bedside blood test results and point-of-care ultrasound findings
- all interventions (investigations and treatments) performed during the patient's management in the ER
- whether the physician was a specialist or resident, and years of experience
- final hospital diagnosis pertaining to the presentation in the ER

Figure 1 summarizes data acquisition during the study period. The primary investigator alone has access to the encryption key. All investigators have access to the anonymized dataset for analysis purposes.

Impact of Emergency Manual use on resuscitation team performance

The final 50 cases where the EM was used in Do-Confirm mode and interventions were performed thanks to checklist access are identified for external review. The final cases during the six-month study period are selected to allow personnel the opportunity to integrate the

For peer review only - http://bmjopen.bmj.com/site/about/guidelines.xhtml

new tool within their clinical practice. The degree to which each intervention is indicated is independently assessed by three specialists in emergency medicine. All three assessors are blinded to whether the intervention was performed before or after EM use. Two assessors are blinded to the content of the EM, while one is involved in the development and use of the EM. Interventions are categorized, based on the available extracted clinical information, as indicated, of neutral relevance, or not indicated. When the three assessors are not in agreement regarding the degree of indication of the intervention, resolution is reached through discussion, and based on majority opinion if no consensus can be reached. Management is deemed improved when interventions categorized as indicated are carried out thanks to EM use. EM use is deemed to not add value if additional interventions are categorized as of neutral relevance. EM use is deemed detrimental if additional interventions are categorized as not indicated.

Sample size calculation

There are no published data on EM use during patient management in the ED. We derived a sample of size of 50 patients where at least one intervention was performed thanks to Do-Confirm EM use based on the following assumptions:

- Among patients with at least one intervention performed thanks to the checklist, half of these patients will have one such intervention performed while the other half will have two. Hence, 50 such patients should generate 25 + 2×25 = 75 interventions thanks to the checklist.
- When multiple checklist-indicated interventions occur in the same patient, the probability of occurrence of these interventions are independent of each other.

In our opinion, a clinically meaningful rate for indicated interventions performed thanks to checklist access is ≥ 10%. Figure 2 shows that if the true rate of clinically indicated

For peer review only - http://bmjopen.bmj.com/site/about/guidelines.xhtml

interventions due to the checklist is ≥ 21% then 50 patients generating 75 interventions will be sufficient to reject null hypotheses that the indicated rate is ≤ 10% with at least 80% power and a Type I error probability of $\alpha \leq 0.05$.

Unperformed interventions recommended by the Emergency Manual

Fifty additional patients where the EM was not used during the pilot study period, and 50 additional patients that presented during the six-month period preceding EM implementation, are also identified. These 100 patients are selected based on matching to the 50 patients where the EM was used using age, presenting complaint and relevant co-morbidities. The EM is accessed to determine whether additional interventions would have been performed, had the EM been used. All interventions—those actually performed as well as those that might have been performed had the EM been used—are then assessed for degree of indication by three external reviewers as described above. The three specialists are blinded to which interventions were actually performed. Analysing this data may suggest that the EM would have improved management had it been used, with the important caveat that many factors that impact on the suitability of interventions may not be fully conveyed in the patient's chart.

Specific case descriptions

The quantitative analyses described above are designed to measure EM added value when used in Do-Confirm mode. Yet EMs are arguably designed to be used in Read-Do or Sampling mode in the setting of a true crisis. EM use may also have unforeseen impacts on patient care and team dynamics that warrant prompt remedying. Resuscitations team personnel are encouraged to request prompt contact—via the digital EM—with the research team to report events or concerns relating to EM use during specific situations. The following

For peer review only - http://bmjopen.bmj.com/site/about/guidelines.xhtml

information is extracted from a structured interview with the personnel regarding the report (Appendix A):

- patient age, sex, presenting complaint and suspected diagnosis
- seniority of the physician initially in charge of the case
- events or concerns relating to EM use
- mode of EM use (Do-Confirm, Read-Do, Sampling) and sections relevant to the case
- personnel's assessment of the impact of the EM on patient care
- personnel's assessment of impact of the EM on team members and teamwork
- personnel's suggestions for EM improvement

Data analysis

Descriptive statistics are used to analyse EM use data and determine the following (average over and trends throughout the pilot study period):

- frequency of EM use and mode of use (Do-Confirm, Read-Do, or Sampling)
- which checklists or fact sheets are most frequently accessed
- time between login to and logout from the digital EM
- subjective degree of EM value and correlation with specific checklists or fact sheets

Descriptive statistics are also used to determine whether EM use is associated with length of stay in the ER, length of hospital admission and mortality. These analyses are hypothesis-generating and not intended to infer causality. For the 150 patients that are externally reviewed, descriptive statistics are used to report the proportions of interventions deemed indicated, of neutral value or not indicated.

For peer review only - http://bmjopen.bmj.com/site/about/guidelines.xhtml

Patient and public involvement

Patients or the public were not involved in the design of this study, nor will they be involved in its conduct or in the reporting and dissemination of our research.

ETHICS AND DISSEMINATION

Ethical and safety considerations

The published literature strongly supports the claim that checklists, used as cognitive aids and not as substitutes for sound clinical judgement, improve performance. Several authors have hypothesized that checklist use may lead to distraction, delays in proper management or wrong management if the wrong checklist is used [22, 42, 43]. However, published studies have not substantiated these concerns. A simulation-based trial of crisis checklists in Lund's ED did not find any delay in the initiation of patient treatment, and all but one of 15 dangerous or inappropriate interventions were carried out by teams without checklist access [21]. One study reported that EMs caused distraction during a small proportion of perioperative crises without negatively impacting on patient care [5]. According to the study protocol, the physician in charge of patient management has the mandate to terminate EM use at any time, and no constraints are imposed on how the EM is used. For these reasons, patient risk incurred from EM access is deemed minimal.

Priority 1 patients are as a rule severely impaired by an acute critical condition and not in a state where informed consent is possible or appropriate to obtain. Written information about EM use will be available in Lund's ER and provided to patients and relatives upon request. Posters informing patients and their relatives about the on-going trial and how to request non-inclusion will be placed throughout the ED. Patient identification numbers are

For peer review only - http://bmjopen.bmj.com/site/about/guidelines.xhtml

pseudonymized automatically and data stored on a computer within the hospital's firewall and locked in a room.

No identifying features of the personnel composing the resuscitation teams are registered during the study, aside from the identity of the personnel logging into the digital EM. Names of personnel involved in the care of specific patients are not recorded during the structured interviews. In the study that evaluated checklists in simulated critical conditions in Lund's ER, over 90% of the care staff stated that they would use the checklist in real crises [21]. Specific reviews of patient management will be based on information available in the medical charts, with the understanding that these cannot convey all relevant factors affecting clinical decisions. No formal complaints or chart reviews will be triggered by the case reviews in this study. For these reasons, risks incurred to resuscitation team personnel from EM access are deemed minimal. The Swedish Ethical Review Authority has approved the study (Dnr 2022-01896-01).

Dissemination plan

Results will be published in a peer-reviewed journal and abstracts submitted to national and international conferences. The simulation-based study of crisis checklists in four EDs in the Skåne Region reported that > 90% of staff at all sites would welcome checklist access during clinical care [21]. Depending on the study results, a multicentre study using customized EMs will be considered.

STRENGTHS, LIMITATIONS & SIGNIFICANCE

The literature on medical crisis checklists argues that specific checklists, used according to clear protocols within specific contexts, improve performance. Yet there are no published

For peer review only - http://bmjopen.bmj.com/site/about/guidelines.xhtml

data on how the introduction of an EM affects the performance of resuscitation teams during the management of actual priority 1 patients in the ED. The main strength of this study is that it evaluates EM use during the management of consecutive, unselected priority 1 patients by resuscitation teams in the ED.

In this study, some of the data on patient characteristics (such as age, chief complaint) and EM use (duration of use, checklists and fact sheets displayed) are automatically registered. Other EM use data (for instance subjective team evaluation of the EM value, modality of EM use) are actively registered by the documentation nurse. The study is not a randomized controlled trial of EM use, and whether the EM is used or not is at the discretion of each resuscitation team. While these study aspects limit the evaluation of how routine EM use would impact on performance, the study will provide information about the circumstances under which resuscitation teams voluntarily use the EM and deem that it adds value to clinical practice. The study stretches over a six-month period, allowing for an analysis of how resuscitation teams integrate a new tool within their clinical practice over time.

The study is not powered nor designed to detect causal relationship between EM use and patient outcome. The study will determine whether interventions carried out thanks to checklist use are indicated, of neutral relevance or not indicated based on the clinical context, as assessed by external reviewers. A limitation of this assessment stems from the fact that the external review is based on information available from patient records, which may not convey all the relevant factors affecting clinical decisions.

This study will be conducted in a single academic Swedish ED, limiting the external validity of the results. Yes this study will provide valuable information to guide the development of a

For peer review only - http://bmjopen.bmj.com/site/about/guidelines.xhtml

tool that has the potential to improve the management of critical patients, and pave the way to multicenter studies of EM use in the ED.

COMPETING INTERESTS STATEMENT

Non declared.

AUTHORS' CONTRIBUTIONS

Eric Dryver conceived of, drafted the original version of, and revised the protocol.

Pontus Olsson de Capretz reviewed and revised the protocol.

Mohammed Mohammad reviewed and revised the protocol.

Malin Armelin reviewed and revised the protocol.

William D Dupont performed the sample size calculations, reviewed and revised the protocol.

Anders Bergenfelz reviewed and revised the protocol.

Ulf Ekelund conceived of, reviewed and revised the protocol.

FUNDING STATEMENT

This work was supported by an ALF research grant (2018-0152) at Skåne University Hospital and by a grant from Region Skåne (2020-0305). This study was part of the AIR Lund (Artificially Intelligent use of Registers at Lund University) research environment and received funding from the Swedish Research Council (VR; grant no. 2019-00198). There was no industry involvement. Funding organizations had no role in the planning, design or realization of the study, collection, analysis or interpretation of data, or preparation, review, or approval of the manuscript.

For peer review only - http://bmjopen.bmj.com/site/about/guidelines.xhtml

Figure 1 Legend

This figure illustrates the two phases of the study, before and after emergency manual implementation, and how data acquisition corresponds to each phase.

Figure 2 Legend

"Intervention" refers here to interventions performed by the resuscitation teams thanks to use of the EM. Interventions are graded by external assessors as indicated, neutral or not indicated based on the clinical context. The power curves in the figure plot the power to determine, with $P \leq 0.05$, that the proportion of interventions deemed indicated exceeds 10% as a function of the true probability that the intervention is indicated. Assuming that the true proportion of interventions deemed indicated is 21%, 75 interventions are required to meet these requirements. Assuming that half of the patients feature one such intervention and the other half two, then 50 paitents can be expected to generate 75 interventions.

REFERENCES

1. Goldhaber-Fiebert, S.N. and S.K. Howard, Implementing emergency manuals: can cognitive aids help translate best practices for patient care during acute events? *Anesth Analg* 2013;117(5):1149-61.
2. Gawande, A., The checklist manifesto : how to get things right. 2010, New York, N.Y., London: Metropolitan Books, Profile Books.
3. Birkmeyer, J.D., Strategies for improving surgical quality--checklists and beyond. *N Engl J Med* 2010;363(20):1963-5.
4. Hearns, S., Checklists in emergency medicine. *Emergency Medicine Journal* 2018;35(9):530-531.
5. Goldhaber-Fiebert, S.N., S. Bereknyei Merrell, A.V. Agarwala, et al., Clinical Uses and Impacts of Emergency Manuals During Perioperative Crises. *Anesth Analg* 2020;131(6):1815-1826.
6. Burian, B.K., A. Clebone, K. Dismukes, et al., More Than a Tick Box: Medical Checklist Development, Design, and Use. *Anesth Analg* 2018;126(1):223-232.
7. Clebone, A., B.K. Burian, and A. Tung, Matching design to use: a task analysis comparison of three cognitive aid designs used during simulated crisis management. *Can J Anaesth* 2019;66(6):658-671.
8. Verdaasdonk, E.G., L.P. Stassen, P.P. Widhiasmara, et al., Requirements for the design and implementation of checklists for surgical processes. *Surg Endosc* 2009;23(4):715-26.

For peer review only - http://bmjopen.bmj.com/site/about/guidelines.xhtml

9. Haynes, A.B., T.G. Weiser, W.R. Berry, et al., A surgical safety checklist to reduce morbidity and mortality in a global population. *N Engl J Med* 2009;360(5):491-9.
10. de Vries, E.N., H.A. Prins, R.M. Crolla, et al., Effect of a comprehensive surgical safety system on patient outcomes. *N Engl J Med* 2010;363(20):1928-37.
11. Sotto, K.T., B.K. Burian, and M. Brindle, Impact of the WHO Surgical Safety Checklist Relative to Its Design and Intended Use: A Systematic Review and Meta-Meta-Analysis. *J Am Coll Surg* 2021.
12. Pronovost, P., D. Needham, S. Berenholtz, et al., An intervention to decrease catheter-related bloodstream infections in the ICU. *N Engl J Med* 2006;355(26):2725-32.
13. Pronovost, P.J., C.A. Goeschel, E. Colantuoni, et al., Sustaining reductions in catheter related bloodstream infections in Michigan intensive care units: observational study. *BMJ* 2010;340:c309.
14. Bion, J., A. Richardson, P. Hibbert, et al., 'Matching Michigan': a 2-year stepped interventional programme to minimise central venous catheter-blood stream infections in intensive care units in England. *BMJ Qual Saf* 2013;22(2):110-23.
15. LeBlanc, V.R., The effects of acute stress on performance: implications for health professions education. *Acad Med* 2009;84(10 Suppl):S25-33.
16. Dismukes, R.K., T.E. Goldsmith, and J.A. Kochan *Effects of acute stress on aircrew performance: literature review and analysis of operational aspects*. 2015.
17. Arriaga, A.F., A.M. Bader, J.M. Wong, et al., Simulation-based trial of surgical-crisis checklists. *N Engl J Med* 2013;368(3):246-53.
18. Just, K.S., S. Hubrich, D. Schmidtke, et al., The effectiveness of an intensive care quick reference checklist manual--a randomized simulation-based trial. *J Crit Care* 2015;30(2):255-60.
19. Hall, C., D. Robertson, M. Rolfe, et al., Do cognitive aids reduce error rates in resuscitation team performance? Trial of emergency medicine protocols in simulation training (TEMPIST) in Australia. *Hum Resour Health* 2020;18(1):1.
20. Knoche, B.B., C. Busche, M. Grodd, et al., A simulation-based pilot study of crisis checklists in the emergency department. *Intern Emerg Med* 2021.
21. Dryver, E., J. Lundager Forberg, C. Hård Af Segerstad, et al., Medical crisis checklists in the emergency department: a simulation-based multi-institutional randomised controlled trial. *BMJ Qual Saf* 2021;30(9):697-705.
22. Simmons, W.R. and J. Huang, Operating Room Emergency Manuals Improve Patient Safety: A Systemic Review. *Cureus* 2019;11(6):e4888.
23. Ranganathan, P., J.H. Phillips, A.F. Attaallah, et al., The use of cognitive aid checklist leading to successful treatment of malignant hyperthermia in an infant undergoing cranioplasty. *Anesth Analg* 2014;118(6):1387.
24. Bereknyei Merrell, S., D.M. Gaba, A.V. Agarwala, et al., Use of an Emergency Manual During an Intraoperative Cardiac Arrest by an Interprofessional Team: A Positive-Exemplar Case Study of a New Patient Safety Tool. *Jt Comm J Qual Patient Saf* 2018;44(8):477-484.
25. Huang, J., F. Jiang, J.-F. Zhang, et al., The use of emergency manuals leading to successful treatment of severe bronchospasm. *The Journal of Medical Practice Management: MPM* 2018;33(5):330-332.
26. Carayon, P., T.B. Wetterneck, A.J. Rivera-Rodriguez, et al., Human factors systems approach to healthcare quality and patient safety. *Appl Ergon* 2014;45(1):14-25.
27. Luedi, M.M. and M.B. Weinger, Emergency Manuals in Context: One Component of Resilient Performance. *Anesthesia & Analgesia* 2020;131(6):1812-1814.

For peer review only - http://bmjopen.bmj.com/site/about/guidelines.xhtml

28. Goldhaber-Fiebert, S.N. and C. Macrae, Emergency Manuals: How Quality Improvement and Implementation Science Can Enable Better Perioperative Management During Crises. *Anesthesiol Clin* 2018;36(1):45-62.
29. Burden, A.R., Z.J. Carr, G.W. Staman, et al., Does every code need a "reader?" improvement of rare event management with a cognitive aid "reader" during a simulated emergency: a pilot study. *Simul Healthc* 2012;7(1):1-9.
30. World Health Organization & WHO Patient Safety, *Implementation Manual - WHO Surgical Safety Checklist (First Edition)*. 2008, World Health Organization: https://apps.who.int/iris/handle/10665/70046.
31. Härtel, C., G. Prosen, R. Brown, et al., *European Core Curriculum for Emergency Medicine. Version 2.0*. 2019 [cited 27 july 2019], European Society for Emergency Medicine: https://eusem.org/images/Curriculum_2.0_WEB.pdf.
32. Civil Aviation Authority (UK) *CAP 708: Guidance on the Design, Presentation and Use of Electronic Checklists*. 2005.
33. Civil Aviation Authority (UK) *CAP 676: Guidance on the Design, Presentation and Use of Emergency and Abnormal Checklists*. 2006.
34. Burian, B.K., Design Guidance for Emergency and Abnormal Checklists in Aviation. *Proceedings of the Human Factors and Ergonomics Society Annual Meeting* 2006;50(1):106-110.
35. Hales, B., M. Terblanche, R. Fowler, et al., Development of medical checklists for improved quality of patient care. *Int J Qual Health Care* 2008;20(1):22-30.
36. Hepner, D.L., A.F. Arriaga, J.B. Cooper, et al., Operating Room Crisis Checklists and Emergency Manuals. *Anesthesiology* 2017;127(2):384-392.
37. Clebone, A., B.K. Burian, and A. Tung, The effect of cognitive aid design on the perceived usability of critical event cognitive aids. *Acta Anaesthesiol Scand* 2020;64(3):378-384.
38. Dryver, E., Emergency Medicine Checklist Compendium. 2022: Media-Tryck, Lund University.
39. Borshoff, D. and S. Weingart, The Resuscitation Crisis Manual. 2018: Leeuwin Press.
40. Goldhaber-Fiebert, S.N., N. Austin, E. Sultan, et al., *Emergency Manual: Cognitive aids for perioperative crises*. 2021: http://emergencymanual.stanford.edu
41. Robertson, D., *Emergency Protocols*, C. Hall, J. Mackenzie, and L. Murray, Editors. 2021: http://emergencyprotocols.org.au.
42. Borshoff, D., The limitations of crisis checklists. *Anesth Analg* 2014;118(6):1387-8.
43. Sollid, S.J.M. and A. Kämäräinen, The checklist, your friend or foe? *Acta Anaesthesiol Scand* 2020;64(1):4-5.

For peer review only - http://bmjopen.bmj.com/site/about/guidelines.xhtml

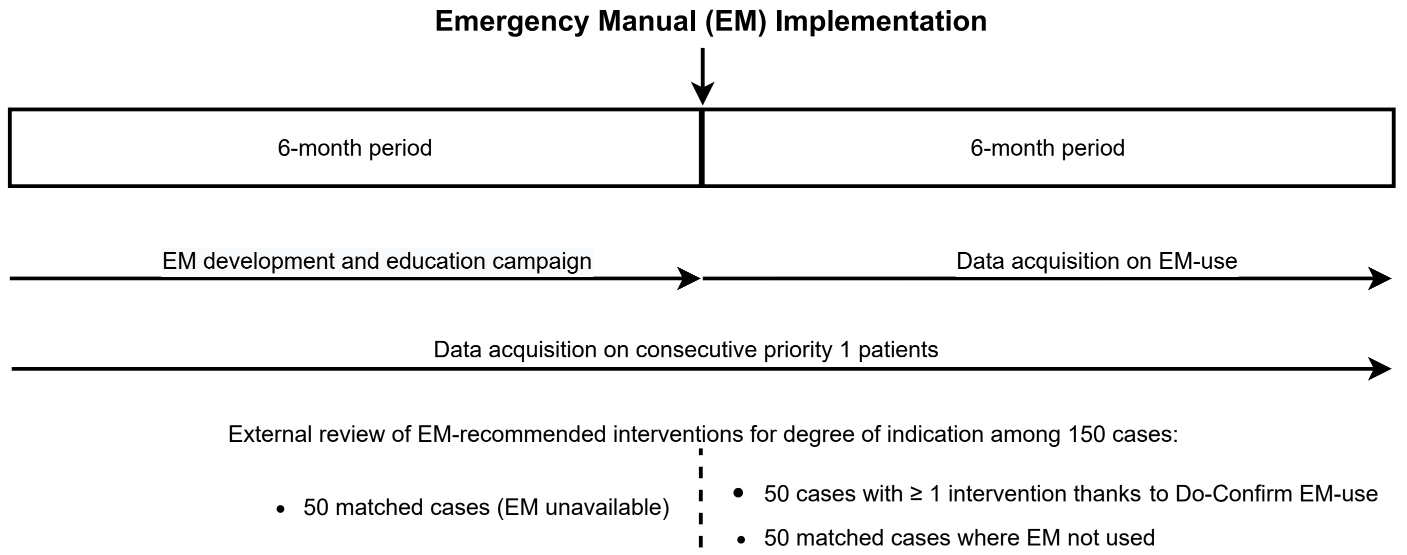
Emergency Manual (EM) Implementation
6-month period
6-month period
EM development and education campaign
Data acquisition on EM-use
Data acquisition on consecutive priority 1 patients
External review of EM-recommended interventions for degree of indication among 150 cases:
50 matched cases (EM unavailable)
50 cases with ≥ 1 intervention thanks to Do-Confirm EM-use
50 matched cases where EM not used

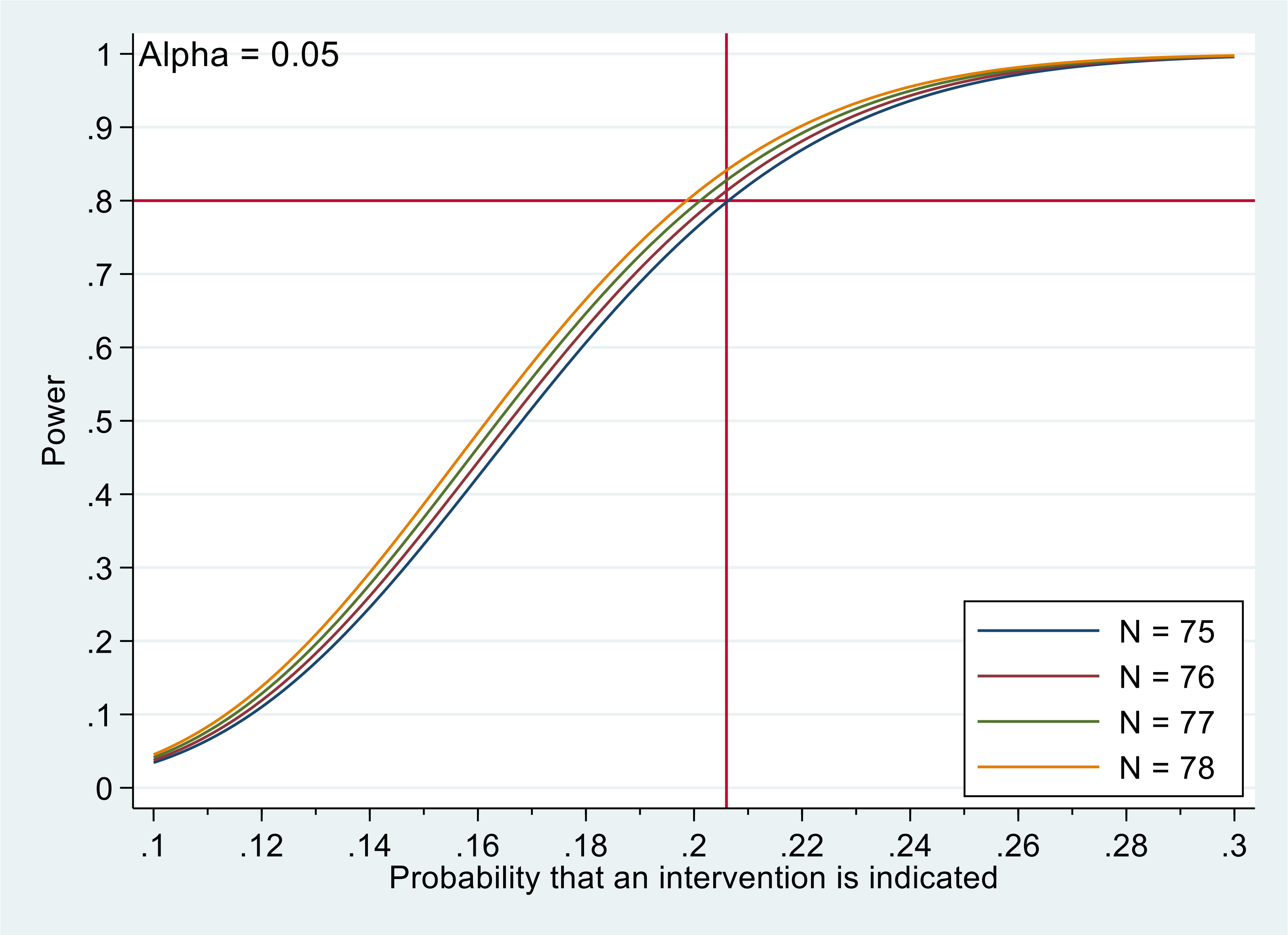
Alpha = 0.05
Power
1
.9
.8
.7
.6
.5
.4
.3
.2
.1
0
.1
.12
.14
.16
.18
.2
.22
.24
.26
.28
.3
Probability that an intervention is indicated
N = 75
N = 76
N = 77
N = 78

Appendix A: Structured Interview Guide for Reported Specific Cases

Introduction: You have requested to speak with us about the use of the Emergency Manual during the management of a specific patient in the resuscitation room. We are interested in understanding how the Emergency Manual is used, how it impacts on actual clinical care and how it can be improved.

Important information:
This project has been approved by the Swedish Ethical Review Authority
The names of specific health care personnel are not recorded.
This information obtained through this interview will not be used for any institutional process for assessing clinical management.

Background Information

Date of the interview:	
Date specific case managed:	
Interviewee's profession:	☐ Nursing assistant ☐ Nurse ☐ Resident ☐ Specialist. Physician's specialty:
Physician in charge of case	☐ Resident ☐ Specialist. Specialty:
Patient age and sex	____________ years. ☐ Male ☐ Female
Presenting complaint	
Suspected diagnosis	

Events or concerns relating to EM use (free text summary, professions of individuals recorded but names NOT recorded)

Emergency Manual Use

Sections of EM relevant to the event	
Mode of EM use	☐ Do-Confirm ☐ Read-Do ☐ Sampling

For peer review only

Assessed impact of EM use on patient care (clinical decisions regarding diagnosis, treatment; free text summary)

Summary of Impact of EM use on patient care: ☐ Negative ☐ Positive

Assessed impact of EM use on team members/teamwork (organization, communication; free text summary)

Summary of Impact of EM use on team members/teamwork: ☐ Negative ☐ Positive

Verbatim summary of how the impact of EM access on the specific case:

Suggestions for improvement (free text summary):

For peer review only

Dnr 2022-01896-01

Göteborg avdelning 2 medicin

BESLUT

2022-04-25

Sökande forskningshuvudman
Region Skåne

Forskare som genomför projektet
Eric Dryver

Projekttitel
Utvärdering av checklistor vid omhändertagande av larmpatienter på akutmottagningen

Uppgifter om ansökan
Ansökan inkom till Etikprövningsmyndigheten 2022-03-31 och blev valid 2022-04-06.

Etikprövningsmyndigheten beslutar enligt nedan.

BESLUT

Etikprövningsmyndigheten godkänner den forskning som anges i ansökan.

På Etikprövningsmyndighetens vägnar

Kavita Bäck Mirchandani
Ordförande

Beslutet har fattats av följande personer:

Ordförande
Kavita Bäck Mirchandani (rådman)

Ledamöter med vetenskaplig kompetens
Karin Manhem (kardiovaskulär sjukdom, prevention, vetenskaplig sekreterare)
Bodil Lernfelt (geriatrik, internmedicin, läkemedel, föredragande)
Bengt Bergman (lungcancer, läkemedelsbehandling, livskvalitet)
Gunnar Göthberg (barnkirurgi med inriktning mage-tarm, pediatrik, kirurgi, vetenskaplig sekreterare Göteborg avdelning 1 medicin)
Linus Jönsson (pediatrik)
Alexandra Krettek (kardiovaskulär prevention, folkhälsovetenskap, biomedicinsk experimentell forskning)
Carmela Miniscalco Mattsson (logopedi, neuropsykiatri, psykologi)

Ledamöter som företräder allmänna intressen
Eleonore Gren
Bengt-Arne Reinholdson
Stig-Olov Tingbratt

Etikprövningsmyndigheten
2022-01896-01-264716
2022-05-02

For peer review only - http://bmjopen.bmj.com/site/about/guidelines.xhtml

Beslutet sänds till

Ansvarig forskare: Eric Dryver

Forskningshuvudmannens företrädare: Oskar Hammar

Etikprövningsmyndigheten
2022-01896-01-264716
2022-05-02

Reporting checklist for protocol of a clinical trial.

Based on the SPIRIT guidelines.

Instructions to authors

Complete this checklist by entering the page numbers from your manuscript where readers will find each of the items listed below.

Your article may not currently address all the items on the checklist. Please modify your text to include the missing information. If you are certain that an item does not apply, please write "n/a" and provide a short explanation.

Upload your completed checklist as an extra file when you submit to a journal.

In your methods section, say that you used the SPIRITreporting guidelines, and cite them as:

Chan A-W, Tetzlaff JM, Gøtzsche PC, Altman DG, Mann H, Berlin J, Dickersin K, Hróbjartsson A, Schulz KF, Parulekar WR, Krleža-Jerić K, Laupacis A, Moher D. SPIRIT 2013 Explanation and Elaboration: Guidance for protocols of clinical trials. BMJ. 2013;346:e7586

		Reporting Item	Page Number
**Administrative information**			
Title	#1	Descriptive title identifying the study design, population, interventions, and, if applicable, trial acronym	1
Trial registration	#2a	Trial identifier and registry name. If not yet registered, name of intended registry	3
Trial registration: data set	#2b	All items from the World Health Organization Trial Registration Data Set	n/a (used registry: clinicaltrials.gov)
Protocol version	#3	Date and version identifier	n/a (version 1)
Funding	#4	Sources and types of financial, material, and other support	20
Roles and responsibilities: contributorship	#5a	Names, affiliations, and roles of protocol contributors	1

For peer review only

Roles and responsibilities: sponsor contact information	#5b	Name and contact information for the trial sponsor	1
Roles and responsibilities: sponsor and funder	#5c	Role of study sponsor and funders, if any, in study design; collection, management, analysis, and interpretation of data; writing of the report; and the decision to submit the report for publication, including whether they will have ultimate authority over any of these activities	20
Roles and responsibilities: committees	#5d	Composition, roles, and responsibilities of the coordinating centre, steering committee, endpoint adjudication committee, data management team, and other individuals or groups overseeing the trial, if applicable (see Item 21a for data monitoring committee)	n/a
**Introduction**			
Background and rationale	#6a	Description of research question and justification for undertaking the trial, including summary of relevant studies (published and unpublished) examining benefits and harms for each intervention	5
Background and rationale: choice of comparators	#6b	Explanation for choice of comparators	n/a (the comparison is management with vs without access to an EM)
Objectives	#7	Specific objectives or hypotheses	8
Trial design	#8	Description of trial design including type of trial (eg, parallel group, crossover, factorial, single group), allocation ratio, and framework (eg, superiority, equivalence, non-inferiority, exploratory)	9
**Methods: Participants,**			

For peer review only - http://bmjopen.bmj.com/site/about/guidelines.xhtml

**interventions, and outcomes**			
Study setting	#9	Description of study settings (eg, community clinic, academic hospital) and list of countries where data will be collected. Reference to where list of study sites can be obtained	9
Eligibility criteria	#10	Inclusion and exclusion criteria for participants. If applicable, eligibility criteria for study centres and individuals who will perform the interventions (eg, surgeons, psychotherapists)	9
Interventions: description	#11a	Interventions for each group with sufficient detail to allow replication, including how and when they will be administered	10
Interventions: modifications	#11b	Criteria for discontinuing or modifying allocated interventions for a given trial participant (eg, drug dose change in response to harms, participant request, or improving / worsening disease)	15
Interventions: adherance	#11c	Strategies to improve adherence to intervention protocols, and any procedures for monitoring adherence (eg, drug tablet return; laboratory tests)	11
Interventions: concomitant care	#11d	Relevant concomitant care and interventions that are permitted or prohibited during the trial	11
Outcomes	#12	Primary, secondary, and other outcomes, including the specific measurement variable (eg, systolic blood pressure), analysis metric (eg, change from baseline, final value, time to event), method of aggregation (eg, median, proportion), and time point for each outcome. Explanation of the clinical relevance of chosen efficacy and harm outcomes is strongly recommended	13

For peer review only

Participant timeline	#13	Time schedule of enrolment, interventions (including any run-ins and washouts), assessments, and visits for participants. A schematic diagram is highly recommended (see Figure)	9 + Figure 1
Sample size	#14	Estimated number of participants needed to achieve study objectives and how it was determined, including clinical and statistical assumptions supporting any sample size calculations	14 + Figure 2
Recruitment	#15	Strategies for achieving adequate participant enrolment to reach target sample size	n/a (consecutive priority 1 patients treated in the ED)
**Methods: Assignment of interventions (for controlled trials)**			
Allocation: sequence generation	#16a	Method of generating the allocation sequence (eg, computer-generated random numbers), and list of any factors for stratification. To reduce predictability of a random sequence, details of any planned restriction (eg, blocking) should be provided in a separate document that is unavailable to those who enrol participants or assign interventions	n/a (one arm study)
Allocation concealment mechanism	#16b	Mechanism of implementing the allocation sequence (eg, central telephone; sequentially numbered, opaque, sealed envelopes), describing any steps to conceal the sequence until interventions are assigned	n/a
Allocation: implementation	#16c	Who will generate the allocation sequence, who will enrol participants, and who will assign participants to interventions	n/a

For peer review only

Blinding (masking)	#17a	Who will be blinded after assignment to interventions (eg, trial participants, care providers, outcome assessors, data analysts), and how	14
Blinding (masking): emergency unblinding	#17b	If blinded, circumstances under which unblinding is permissible, and procedure for revealing a participant's allocated intervention during the trial	n/a (no blinding for intervention, i.e. access to checklists)
**Methods: Data collection, management, and analysis**			
Data collection plan	#18a	Plans for assessment and collection of outcome, baseline, and other trial data, including any related processes to promote data quality (eg, duplicate measurements, training of assessors) and a description of study instruments (eg, questionnaires, laboratory tests) along with their reliability and validity, if known. Reference to where data collection forms can be found, if not in the protocol	12
Data collection plan: retention	#18b	Plans to promote participant retention and complete follow-up, including list of any outcome data to be collected for participants who discontinue or deviate from intervention protocols	n/a (consecutive patients management in the ED)
Data management	#19	Plans for data entry, coding, security, and storage, including any related processes to promote data quality (eg, double data entry; range checks for data values). Reference to where details of data management procedures can be found, if not in the protocol	12
Statistics: outcomes	#20a	Statistical methods for analysing primary and secondary outcomes. Reference to	16

For peer review only

		where other details of the statistical analysis plan can be found, if not in the protocol	
Statistics: additional analyses	#20b	Methods for any additional analyses (eg, subgroup and adjusted analyses)	16
Statistics: analysis population and missing data	#20c	Definition of analysis population relating to protocol non-adherence (eg, as randomised analysis), and any statistical methods to handle missing data (eg, multiple imputation)	n/a
**Methods: Monitoring**			
Data monitoring: formal committee	#21a	Composition of data monitoring committee (DMC); summary of its role and reporting structure; statement of whether it is independent from the sponsor and competing interests; and reference to where further details about its charter can be found, if not in the protocol. Alternatively, an explanation of why a DMC is not needed	n/a (checklist use is not mandatory; there is no evidence that checklist access is hazardous to patient management; teams can request immediate contact with the investigators to report specific events associated with checklist use)
Data monitoring: interim analysis	#21b	Description of any interim analyses and stopping guidelines, including who will have access to these interim results and make the final decision to terminate the trial	n/a
Harms	#22	Plans for collecting, assessing, reporting, and managing solicited and spontaneously reported adverse events and other unintended effects of trial interventions or trial conduct	15
Auditing	#23	Frequency and procedures for auditing trial conduct, if any, and whether the process will be independent from investigators and the sponsor	n/a (this study focuses on how teams use a new tool)
**Ethics and dissemination**			
Research ethics	#24	Plans for seeking research ethics committee	18

For peer review only

approval		/ institutional review board (REC / IRB) approval	
Protocol amendments	#25	Plans for communicating important protocol modifications (eg, changes to eligibility criteria, outcomes, analyses) to relevant parties (eg, investigators, REC / IRBs, trial participants, trial registries, journals, regulators)	n/a (consecutive unselected patients managed in the emergency room)
Consent or assent	#26a	Who will obtain informed consent or assent from potential trial participants or authorised surrogates, and how (see Item 32)	17
Consent or assent: ancillary studies	#26b	Additional consent provisions for collection and use of participant data and biological specimens in ancillary studies, if applicable	n/a (no such data)
Confidentiality	#27	How personal information about potential and enrolled participants will be collected, shared, and maintained in order to protect confidentiality before, during, and after the trial	12
Declaration of interests	#28	Financial and other competing interests for principal investigators for the overall trial and each study site	20
Data access	#29	Statement of who will have access to the final trial dataset, and disclosure of contractual agreements that limit such access for investigators	13
Ancillary and post trial care	#30	Provisions, if any, for ancillary and post-trial care, and for compensation to those who suffer harm from trial participation	n/a
Dissemination policy: trial results	#31a	Plans for investigators and sponsor to communicate trial results to participants, healthcare professionals, the public, and other relevant groups (eg, via publication, reporting in results databases, or other data	18

For peer review only

		sharing arrangements), including any publication restrictions	
Dissemination policy: authorship	#31b	Authorship eligibility guidelines and any intended use of professional writers	n/a
Dissemination policy: reproducible research	#31c	Plans, if any, for granting public access to the full protocol, participant-level dataset, and statistical code	n/a
**Appendices**			
Informed consent materials	#32	Model consent form and other related documentation given to participants and authorised surrogates	n/a (see above)
Biological specimens	#33	Plans for collection, laboratory evaluation, and storage of biological specimens for genetic or molecular analysis in the current trial and for future use in ancillary studies, if applicable	n/a (no such data collected)

None The SPIRIT Explanation and Elaboration paper is distributed under the terms of the Creative Commons Attribution License CC-BY-NC. This checklist can be completed online using https://www.goodreports.org/, a tool made by the EQUATOR Network in collaboration with Penelope.ai

For peer review only

BMJ Open

Clinical use of an emergency manual by resuscitation teams and impact on performance in the emergency department—a prospective mixed-methods study protocol

Journal:	*BMJ Open*
Manuscript ID	bmjopen-2022-071545.R1
Article Type:	Protocol
Date Submitted by the Author:	16-Mar-2023
Complete List of Authors:	Dryver, Eric; Skåne University Hospital Lund, Department of Emergency and Internal Medicine; Lund University, Department of Clinical Sciences at Lund<br>Olsson de Capretz, Pontus; Skåne University Hospital Lund, Department of Emergency and Internal Medicine; Lund University, Department of Clinical Sciences at Lund<br>Mohammad, Mohammed; Skåne University Hospital Lund, Department of Emergency and Internal Medicine<br>Armelin, Malin; Skåne University Hospital Lund, Department of Emergency and Internal Medicine<br>Dupont, William D. ; Vanderbilt University Medical Center, Department of Biostatistics<br>Bergenfelz, Anders; Lund University, Department of Clinical Sciences at Lund<br>Ekelund, Ulf; Skåne University Hospital Lund, Department of Emergency and Internal Medicine; Lund University, Department of Clinical Sciences at Lund
**Primary Subject Heading**:	Emergency medicine
Secondary Subject Heading:	Emergency medicine
Keywords:	ACCIDENT & EMERGENCY MEDICINE, INTENSIVE & CRITICAL CARE, Protocols & guidelines < HEALTH SERVICES ADMINISTRATION & MANAGEMENT

SCHOLARONE™ Manuscripts

TITLE PAGE

Clinical use of an emergency manual by resuscitation teams and impact on performance in the emergency department—a prospective mixed-methods study protocol

AUTHORS

Eric Dryver[*†‡], M.D. eric.dryver@med.lu.se
Pontus Olsson de Capretz[*†], M.D. pontus.olsson_de_capretz@med.lu.se
Mohammed Mohammad[*], M.D. mohammed.mohammad@skane.se
Malin Armelin[*], RN, MSN Malin.Armelin@skane.se
William D Dupont[§], Ph.D. william.dupont@vumc.org
Anders Bergenfelz[†], M.D., Ph.D. anders.bergenfelz@med.lu.se
Ulf Ekelund[*†], M.D., Ph.D. ulf.ekelund@med.lu.se

* Department of Emergency and Internal Medicine, Skåne University Hospital at Lund, Sweden
† Department of Clinical Sciences at Lund, Lund University, Sweden
‡ Practicum Clinical Skills Centre, Lund, Sweden
§ Department of Biostatistics, Vanderbilt University Medical Center, USA

CORRESPONDING AUTHOR

Eric Dryver
Department of Emergency Medicine
Skåne University Hospital at Lund
Klinikgatan 15
22185 Lund
Sweden
eric.dryver@med.lu.se

WORD COUNT

4124

For peer review only - http://bmjopen.bmj.com/site/about/guidelines.xhtml

ABSTRACT

Introduction

Simulation-based studies indicate that crisis checklist use improves management of patients with critical conditions in the emergency department (ED). An interview-based study suggests that use of an emergency manual (EM)—a collection of crisis checklists—improves management of clinical perioperative crises. To date, there are no prospective studies of EMs during clinical practice.

Methods and analysis

This six-month-long study prospectively evaluates a digital EM during management of priority 1 patients in the Skåne University Hospital at Lund's ED. Resuscitation teams are encouraged to use the EM after a management plan has been derived ("Do-Confirm"). The documenting nurse is assigned the role of activating and reading from the EM, and checklists are displayed on a large screen visible to all team-members. Whether the EM is activated, and which sections are displayed, are automatically recorded. Interventions performed thanks to Do-Confirm EM use are registered by the nurse. Fifty cases featuring such interventions are reviewed by specialists in emergency medicine blinded to whether the interventions were performed prior to or after EM use. All interventions are graded as indicated, of neutral relevance or not indicated. The primary outcome measures are the proportions of interventions performed thanks to Do-Confirm EM use graded as indicated, of neutral relevance, and not indicated. A secondary outcome measure is the team's subjective evaluation of the EM's value on a Likert scale of 1 to 6. Team members are encouraged to report events related to EM use, and information from these events are extracted through structured interviews.

For peer review only - http://bmjopen.bmj.com/site/about/guidelines.xhtml

Ethics and dissemination

The study has been approved by the Swedish Ethical Review Authority (Dnr 2022-01896-01). Results will be published in a peer-reviewed journal and abstracts submitted to national and international conferences to disseminate our findings.

Registration

The trial is registered at clinicaltrials.gov (NCT 05649891).

For peer review only

For peer review only - http://bmjopen.bmj.com/site/about/guidelines.xhtml

STRENGTHS AND LIMITATIONS OF THIS STUDY

- To date, studies of Emergency Manuals (EM)—collections of crisis checklists— have been simulation- or interview-based. This study prospectively evaluates an EM during the management of unselected, consecutive priority 1 patients in an emergency department over a six-month period.
- Some of the data on patient characteristics and EM use are automatically registered, while other EM use data are actively registered by the documenting nurse.
- The study aims to determine whether interventions carried out thanks to checklist use are indicated, of neutral relevance or not indicated based on the clinical context, as assessed by external reviewers.
- The external review process is based on information available from patient records, which may not convey all the relevant factors affecting clinical decisions.
- The study is not a randomized-controlled trial and is not powered nor designed to detect causal relationship between EM use and patient outcome.

For peer review only

INTRODUCTION

Checklists

A checklist is a cognitive aid—consisting of a list of assessments or actions—designed to be carried out systematically. The function of checklists is to improve task performance; checklists are essentially knowledge-translation tools that promote completeness, consistency and alignment with best practice guidelines when carrying out a task [1]. Checklists work by directing focus, providing facts [2], and guiding—though do not dictating—decision-making. Checklists are also believed to improve performance through other mechanisms, such as reducing mental workload, promoting a shared mental model among team-members and empowering team-members to voice concerns [3-5].

There are two basic modes of checklist use [6]. In Read-Do mode, checklists are used as the task is performed to guide task completion. During the Do-Confirm mode, checklists are used after task performance to verify completeness. Cognitive aids may even be used to retrieve specific information (for instance the dosage of a specific medication in a specific setting), so-called Sampling mode [6, 7]. Checklists may be used by one or several people, silently or read aloud [6], and can be categorized according to the circumstances in which they are used: normal (or routine) situations, emergency situations, and atypical (or non-normal) situations [6, 8].

Checklists for routine situations in health care

The World Health Organization (WHO) Surgical Safety Checklist [9] and the Surgical Safety Patient System [10] are examples of checklists used to improve performance during routine surgical procedures. There is substantial, robust, international evidence that use of the WHO Surgical Safety Checklist reduces mortality and complications associated with general

For peer review only

surgery [11]. A checklist designed to minimize bloodstream infections associated with the routine insertion of central venous catheters in the intensive care unit is also associated with improved performance during routine health care delivery [12-14].

Checklists for health care emergencies

Stress affects attention and information retrieval from memory [15, 16], and hence checklists may play a particular role in improving performance during stressful emergencies [1, 4]. Due to the unexpected, low-frequency nature of medical emergencies, crisis checklists have mainly been studied using simulation. One study reported a reduction in the frequency of unperformed lifesaving processes of care from 23% to 6% associated with checklist access during simulated surgical crises [17]. Another study reported that checklist use increased the number of key items performed during scenarios in a simulated intensive care unit from 7 to 9 [18]. An additional study performed in a simulated surgical ward reported that use of a cognitive aid reduced the frequency of omitted critical management steps from 33% to 10% [19].

Two studies evaluated crisis checklists in a simulated resuscitation room. One study evaluated the use of an Emergency Protocols Handbook through four scenarios and reported that checklist use decreased error rate from 39% to 19% [20]. The other study evaluated a collection of four checklists through four scenarios; checklist use was associated with an increased frequency of hemodynamic stabilization and diagnosis recognition, as well as an increased adherence to critical steps (56% without checklist, 82% with checklists) [21].

A simulation-based, randomized controlled trial of medical crisis checklists was performed in four resuscitation rooms using actual resuscitation teams during their clinical shifts [22].

For peer review only - http://bmjopen.bmj.com/site/about/guidelines.xhtml

Eight medical crises were simulated. With checklist access, the median percentage of key interventions performed was 86%; without access, the median percentage was 39%.

Evidence that collections of crisis checklists and cognitive aid elements such as medication doses—Emergency Manuals (EM)—improve actual clinical care is limited. Several reports document successful management of perioperative crises thanks to the use of operating room EMs [23-26]. An interview-based study of anaesthesiologists involved in perioperative crises in 69 patients revealed that EM use led to identifying and addressing errors of omission in 59% of cases, while lack of EM use was associated with self-identified errors of omission or delays in intervention performance in 56% of cases [5].

Systematic crisis checklist evaluation in the clinical setting

Performance is the end-product of complex interactions between people, the tools at their disposal, and the physical and cultural environments they work in [27]. Checklists do not work in isolation, and their impact on performance is contingent not only upon their content and design but also on when, where, how and by whom they are used. Simulation-based trials and interview-based studies [28] provide limited evidence for how checklists may actually impact resuscitation team performance in a specific clinical setting. Several authors have called for pilot-studies that systematically evaluate crisis checklists in the clinical setting [5, 19, 20, 22, 28, 29].

Experts emphasize the importance of establishing the rational for EM use, fostering a sense of local EM ownership, ensuring access and familiarity with the tool, and customization [30]. In particular, an explicit protocol is likely required to ensure that checklists are used as intended. A simulation-based study reported that the mere access to crisis checklists did not

For peer review only - http://bmjopen.bmj.com/site/about/guidelines.xhtml

ensure their systematic use; only when an assigned team-member read aloud the steps on the checklist were all critical actions performed [31]. The Surgical Safety Checklist Implementation Manual emphasizes the importance of clearly assigning the role of checklist implementation to a specific person[32] and an interview-based study of EM use during perioperative crises reported beneficial effects of a reader-leader combination [5].

Challenges to implementation and evaluation of an EM in the ED include the following:

- the number of possible presenting complaints, acute conditions and pre-existing co-morbidities among priority 1 patients is large, and the degree of actual acuity is variable
- the number of personnel that work in the resuscitation room is large, and each personnel has variable context-specific degrees of expertise; managing a patient with acute pulmonary edema may be "routine" for a seasoned physician and an "emergency" for a junior resident
- Do-Confirm mode of checklist use is best suited to quantify the added value of checklist use, while Read-Do and Sampling modes may be best suited for checklist use during an actual crisis

These challenges have informed the study design.

Objectives

The primary objective of the study is to determine whether Do-Confirm EM use leads to the performance, by resuscitation teams managing priority 1 patients in the ED, of additional interventions that are deemed indicated, of neutral relevance or not indicated given the clinical context. Secondary study objectives are

- determining the frequency and modality (Do-Confirm, Read-Do, Sampling) with which the EM is used and whether these changed significantly over the study period

For peer review only - http://bmjopen.bmj.com/site/about/guidelines.xhtml

- determining whether Do-Confirm EM use in selected cases where other modes were used might have led to the performance of additional indicated interventions
- determining the degree to which resuscitation teams find the EM helpful, and under which circumstances

METHODS AND ANALYSIS

Setting, patient population and personnel

The study will be carried out in the resuscitation room of the ED of Skåne's University Hospital at Lund (referred to hereafter as Lund's RR). Lund's RR is designed for the simultaneous management of three priority 1 patients. EM access during clinical practice will be provided in September 2023. The study population consists of consecutive priority 1 patients managed in the Lund's RR during the six-month period prior to, and the six-month pilot study period following EM implementation. Patients are deemed to be priority 1 based on vital signs and symptoms according to the Rapid Emergency Triage and Treatment System (http://predicare.se/) the most commonly used triage system in Sweden. The yearly number of priority 1 patients treated in Lund's RR is roughly 4000 and consists of adult patients (> 18 years) with medical or surgical emergencies and patients of all ages with level 1 trauma. Most of these patients are brought to the RR via ambulance, and only occasionally are they identified at the triage or at later stage of ED care. All priority 1 patients are managed immediately in the RR by multidisciplinary resuscitation teams. The core resuscitation team consists of a resident or specialist in emergency medicine, two nurses and one nursing assistant. When the patient is especially critical (for instance cardiac arrest, level 1 trauma, upper airway obstruction), additional personnel (anaesthesiologists, surgeons, cardiologists, otorhinolaryngologists) supplement the resuscitation team.

For peer review only - http://bmjopen.bmj.com/site/about/guidelines.xhtml

Emergency Manual: content and format

The Emergency Manual consists of diagnosis checklists, problem checklists, procedure checklists and fact sheets in digital form. The checklists and fact sheets are accessed through a tablet computer located at each of the three resuscitation sections in the RR and displayed on a large screen visible to all members of the resuscitation team. Diagnosis checklists consist of lists of interventions to consider during the management of a suspected diagnosis. Each intervention is associated with a popover window displaying indications, contraindications or risks associated with the intervention, and details regarding how the intervention is performed, as described in a previous study [22]. Problem checklists consist of lists of interventions and diagnoses to consider based on the patient's presenting symptoms. The popover windows display key clinical features suggestive of specific diagnoses. Procedure checklists provide step-by-step guidance for the performance of key procedures as well as pictures. Fact sheets provide information such as medical dosages for sedation and anaesthesia for patients of all ages.

The collection of checklists and fact sheets covers the majority of symptoms, diagnoses and situations that may present in the RR, in accordance with the current European Core Curriculum in Emergency Medicine [33]. The format and design of each checklist or fact sheet is informed by the literature pertaining to emergency and abnormal checklists in the aviation industry [34-36], articles on medical checklists [6, 37-39] and a selection of EMs [40-43]. The content is customized to local circumstances in Lund's ED (available medications, medication locations, local routines and phone numbers). EM content and format are further developed through iterative, rapid-cycle simulation-based testing and review by specialists, residents, nurses and nursing assistants who work clinically in Lund’s RR. Internet access to the EM is provided to the ED personnel prior to and during the study

For peer review only - http://bmjopen.bmj.com/site/about/guidelines.xhtml

to allow for familiarization and suggestions for improvement. Each checklist and fact sheet is approved by at least one specialist in emergency medicine and one nurse, both of whom work clinically in Lund's RR.

Emergency Manual: protocol for use

EM use during priority 1 management is strongly advocated but not mandatory. The documenting nurse is assigned the specific task of activating the digital EM. We recommend Do-Confirm as the default mode of EM use—the team first manages priority 1 patients without support from the EM. The documenting nurse then asks the team: "Which checklist should I display?" When a checklist is displayed, the documenting nurse reads aloud each potential intervention/diagnosis for team consideration. The documenting team-member registers on the digital checklist whether each intervention is:

- deemed indicated and has already been carried out
- deemed irrelevant or contraindicated
- deemed indicated and will be carried out thanks to EM use

The physician responsible for patient management has the mandate to request that the EM not be used, to pause or terminate EM use at any time, and to depart from the recommendations featuring on the checklist. Physicians may also request that the EM be displayed directly and used in Read-Do mode or for retrieval of specific information (Sampling mode). After EM use, teams grade the usefulness of the EM on a 1-6 Likert scale prior to logging out. If the documenting nurse logs in to the EM but the EM is not used, the nurse selects among potential reasons for non-use prior to logging out. Teams can also electronically request contact with the investigators to report specific events related to EM use.

For peer review only

For peer review only - http://bmjopen.bmj.com/site/about/guidelines.xhtml

Educational campaign

An educational campaign targeting all personnel who work in Lund's RR precedes the implementation of the EM in clinical practice. Evidence for the potential benefits of an EM—including results from a locally-performed crisis checklist study [22]—is presented, as well as the protocol for EM use. Documenting nurses during priority 1 patient management are taught to log in to the EM, display and navigate between checklists, open popover windows, record whether interventions were not indicated, done before or thanks to checklist access, record the team's opinion of the EM's value, request contact with the investigators, and log out. Documenting nurses carry out these functions in addition to their usual documentation tasks.

Data acquisition

The following data pertaining to each priority 1 patient managed during the six-month period prior to, and the six-month pilot study period following implementation of the EM in clinical practice, are extracted from Region Skåne's clinical databases and the national data registry SVAR (Svenska Akutvårdsregistret):

- patient age and sex, presenting complaint and vital signs
- presumptive diagnosis in the RR
- time spent in the RR, in the ED, and admitted to the hospital
- whether the patient was admitted to the intensive care unit, and if so duration of admission
- seven-day and thirty-day mortality

The following data regarding EM use are recorded on a dedicated computer located within the hospital's firewall each time the EM is activated during the six-month pilot study period:

- log-in and log-out times

For peer review only - http://bmjopen.bmj.com/site/about/guidelines.xhtml

- identification number of the nurse who logged in, level of the most senior physician who was physically present at the bedside during patient management, and patient identification number (which is immediately encrypted)
- checklists and fact sheets that were displayed
- whether interventions were performed before or thanks to checklist access when the EM was used in Do-Confirm mode, or whether the EM was used in Read-Do or Sampling mode instead
- the team's subjective evaluation of the value of the EM according to a 1-6 Likert scale
- reasons for EM non-use despite logging in
- whether prompt contact with the research team is requested

The following data is extracted from the medical records of 150 selected cases (see below):

- baseline information regarding the patient's age, sex, known medical background at the time of presentation and current medications
- clinical information regarding the patient's presentation to the RR: ambulance report, history that can be obtained in the RR, vital signs, physical findings, electrocardiogram, bedside blood test results and point-of-care ultrasound findings
- all interventions (investigations and treatments) performed during the patient's management in the RR
- whether the physician was a specialist or resident, and years of experience
- final hospital diagnosis pertaining to the presentation in the RR

Figure 1 summarizes data acquisition during the study period. The primary investigator alone has access to the encryption key. All investigators have access to the anonymized dataset for analysis purposes.

For peer review only

Impact of Emergency Manual use on resuscitation team performance

The final 50 cases where the EM was used in Do-Confirm mode and interventions were performed thanks to checklist access are identified for external review. The final cases during the six-month study period are selected to allow personnel the opportunity to integrate the new tool within their clinical practice. The degree to which each intervention is indicated is independently assessed by three specialists in emergency medicine. All three assessors are blinded to whether the intervention was performed before or after EM use. Two assessors are blinded to the content of the EM, while one is involved in the development and use of the EM. Interventions are categorized, based on the available extracted clinical information, as indicated (e.g. administering antibiotics to the septic patient), of neutral relevance (e.g. measuring liver function tests for the patient with suspected benzodiazepine overdose), or not indicated (i.e. over-investigation or -treatment, e.g. ordering computed tomography to rule out pulmonary embolism for a patient with pulmonary edema). When the three assessors are not in agreement regarding the degree of indication of the intervention, resolution is reached through discussion, and based on majority opinion if no consensus can be reached. Management is deemed improved when interventions categorized as indicated are carried out thanks to EM use. EM use is deemed to not add value if additional interventions are categorized as of neutral relevance. EM use is deemed detrimental if additional interventions are categorized as not indicated.

Sample size calculation

There are no published data on EM use during patient management in the ED. We derived a sample of size of 50 patients where at least one intervention was performed thanks to Do-Confirm EM use based on the following assumptions:

For peer review only - http://bmjopen.bmj.com/site/about/guidelines.xhtml

- Among patients with at least one intervention performed thanks to the checklist, half of these patients will have one such intervention performed while the other half will have two. Hence, 50 such patients should generate 25 + 2×25 = 75 interventions thanks to the checklist.
- When multiple checklist-indicated interventions occur in the same patient, the probability of occurrence of these interventions are independent of each other.

In our opinion, a clinically meaningful rate for indicated interventions performed thanks to checklist access is ≥ 10%. Figure 2 shows that if the true rate of clinically indicated interventions due to the checklist is ≥ 21% then 50 patients generating 75 interventions will be sufficient to reject null hypotheses that the indicated rate is ≤ 10% with at least 80% power and a Type I error probability of $\alpha \leq 0.05$.

Unperformed interventions recommended by the Emergency Manual

Fifty additional patients where the EM was not used during the pilot study period, and 50 additional patients that presented during the six-month period preceding EM implementation, are also identified. These 100 patients are selected based on matching to the 50 patients where the EM was used using age, presenting complaint and relevant co-morbidities. The EM is accessed to determine whether additional interventions would have been performed, had the EM been used. All interventions—those actually performed as well as those that might have been performed had the EM been used—are then assessed for degree of indication by three external reviewers as described above. The three specialists are blinded to which interventions were actually performed. Analysing this data may suggest that the EM would have improved management had it been used, with the important caveat that many factors that impact on the suitability of interventions may not be fully conveyed in the patient's chart.

For peer review only

Specific case descriptions

The quantitative analyses described above are designed to measure EM added value when used in Do-Confirm mode. Yet EMs are arguably designed to be used in Read-Do or Sampling mode in the setting of a true crisis. EM use may also have unforeseen impacts on patient care and team dynamics that warrant prompt remedying. Resuscitations team personnel are encouraged to request prompt contact—via the digital EM—with the research team to report events or concerns relating to EM use during specific situations. The following information is extracted from a structured interview with the personnel regarding the report (Appendix A):

- patient age, sex, presenting complaint and suspected diagnosis
- seniority of the physician initially in charge of the case
- events or concerns relating to EM use
- mode of EM use (Do-Confirm, Read-Do, Sampling) and sections relevant to the case
- personnel's assessment of the impact of the EM on patient care
- personnel's assessment of impact of the EM on team members and teamwork
- personnel's suggestions for EM improvement

Data analysis

Descriptive statistics are used to analyse EM use data and determine the following (average over and trends throughout the pilot study period):

- frequency of EM use and mode of use (Do-Confirm, Read-Do, or Sampling)
- which checklists or fact sheets are most frequently accessed
- time between login to and logout from the digital EM
- subjective degree of EM value and correlation with specific checklists or fact sheets

For peer review only - http://bmjopen.bmj.com/site/about/guidelines.xhtml

Descriptive statistics are also used to determine whether EM use is associated with length of stay in the RR, length of hospital admission and mortality. These analyses are hypothesis-generating and not intended to infer causality. For the 150 patients that are externally reviewed, descriptive statistics are used to report the proportions of interventions deemed indicated, of neutral value or not indicated.

Patient and public involvement

Patients or the public were not involved in the design of this study, nor will they be involved in its conduct or in the reporting and dissemination of our research.

ETHICS AND DISSEMINATION

Ethical and safety considerations

The published literature strongly supports the claim that checklists, used as cognitive aids and not as substitutes for sound clinical judgement, improve performance. Several authors have hypothesized that checklist use may lead to distraction, delays in proper management or wrong management if the wrong checklist is used [23, 44, 45]. However, published studies have not substantiated these concerns. A simulation-based trial of crisis checklists in Lund's ED did not find any delay in the initiation of patient treatment, and all but one of 15 dangerous or inappropriate interventions were carried out by teams without checklist access [22]. One study reported that EMs caused distraction during a small proportion of perioperative crises without negatively impacting on patient care [5]. According to the study protocol, the physician in charge of patient management has the mandate to terminate EM use at any time, and no constraints are imposed on how the EM is used. For these reasons, patient risk incurred from EM access is deemed minimal.

For peer review only

Priority 1 patients are as a rule severely impaired by an acute critical condition and not in a state where informed consent is possible or appropriate to obtain. Written information about EM use will be available in Lund's RR and provided to patients and relatives upon request. Posters informing patients and their relatives about the on-going trial and how to request non-inclusion will be placed throughout the ED. Patient identification numbers are pseudonymized automatically and data stored on a computer within the hospital's firewall and locked in a room.

No identifying features of the personnel composing the resuscitation teams are registered during the study, aside from the identity of the personnel logging into the digital EM. Names of personnel involved in the care of specific patients are not recorded during the structured interviews. In the study that evaluated checklists in simulated critical conditions in Lund's RR, over 90% of the care staff stated that they would use the checklist in real crises [22]. Specific reviews of patient management will be based on information available in the medical charts, with the understanding that these cannot convey all relevant factors affecting clinical decisions. No formal complaints or chart reviews will be triggered by the case reviews in this study. For these reasons, risks incurred to resuscitation team personnel from EM access are deemed minimal. The Swedish Ethical Review Authority has approved the study (Dnr 2022-01896-01).

Dissemination plan

Results will be published in a peer-reviewed journal and abstracts submitted to national and international conferences. The simulation-based study of crisis checklists in four EDs in the Skåne Region reported that > 90% of staff at all sites would welcome checklist access during

For peer review only

clinical care [22]. Depending on the study results, a multicentre study using customized EMs will be considered.

STRENGTHS, LIMITATIONS & SIGNIFICANCE

The literature on medical crisis checklists argues that specific checklists, used according to clear protocols within specific contexts, improve performance. Yet there are no published data on how the introduction of an EM affects the performance of resuscitation teams during the management of actual priority 1 patients in the ED. The main strength of this study is that it evaluates EM use during the management of consecutive, unselected priority 1 patients by resuscitation teams in the ED.

In this study, some of the data on patient characteristics (such as age, chief complaint) and EM use (duration of use, checklists and fact sheets displayed) are automatically registered. Other EM use data (for instance subjective team evaluation of the EM value, modality of EM use) are actively registered by the documenting nurse. The study is not a randomized controlled trial of EM use, and whether the EM is used or not is at the discretion of each resuscitation team. While these study aspects limit the evaluation of how routine EM use would impact on performance, the study will provide information about the circumstances under which resuscitation teams voluntarily use the EM and deem that it adds value to clinical practice. The study stretches over a six-month period, allowing for an analysis of how resuscitation teams integrate a new tool within their clinical practice over time.

The study is not powered nor designed to detect causal relationship between EM use and patient outcome. The study will determine whether interventions carried out thanks to checklist use are indicated, of neutral relevance or not indicated based on the clinical context,

For peer review only - http://bmjopen.bmj.com/site/about/guidelines.xhtml

as assessed by external reviewers. A limitation of this assessment stems from the fact that the external review is based on information available from patient records, which may not convey all the relevant factors affecting clinical decisions.

This study will be conducted in a single academic Swedish ED, limiting the external validity of the results. Yet this study will provide valuable information to guide the development of a tool that has the potential to improve the management of critical patients, and pave the way to multicenter studies of EM use in the ED.

COMPETING INTERESTS STATEMENT

Non declared.

AUTHORS' CONTRIBUTIONS

Eric Dryver conceived of, drafted the original version of, and revised the protocol.

Pontus Olsson de Capretz reviewed and revised the protocol.

Mohammed Mohammad reviewed and revised the protocol.

Malin Armelin reviewed and revised the protocol.

William D Dupont performed the sample size calculations, reviewed and revised the protocol.

Anders Bergenfelz reviewed and revised the protocol.

Ulf Ekelund conceived of, reviewed and revised the protocol.

FUNDING STATEMENT

This work was supported by an ALF research grant (2018-0152) at Skåne University Hospital and by a grant from Region Skåne (2020-0305). This study was part of the AIR Lund (Artificially Intelligent use of Registers at Lund University) research environment and

For peer review only - http://bmjopen.bmj.com/site/about/guidelines.xhtml

received funding from the Swedish Research Council (VR; grant no. 2019-00198). There was no industry involvement. Funding organizations had no role in the planning, design or realization of the study, collection, analysis or interpretation of data, or preparation, review, or approval of the manuscript.

For peer review only

Figure 1 Legend

This figure illustrates the two phases of the study, before and after emergency manual implementation, and how data acquisition corresponds to each phase.

Figure 2 Legend

"Intervention" refers here to interventions performed by the resuscitation teams thanks to use of the EM. Interventions are graded by external assessors as indicated, neutral or not indicated based on the clinical context. The power curves in the figure plot the power to determine, with $P \leq 0.05$, that the proportion of interventions deemed indicated exceeds 10% as a function of the true probability that the intervention is indicated. Assuming that the true proportion of interventions deemed indicated is 21%, 75 interventions are required to meet these requirements. Assuming that half of the patients feature one such intervention and the other half two, then 50 patients can be expected to generate 75 interventions.

For peer review only

REFERENCES

1. Goldhaber-Fiebert, S.N. and S.K. Howard, Implementing emergency manuals: can cognitive aids help translate best practices for patient care during acute events? *Anesth Analg* 2013;117(5):1149-61.
2. Gawande, A., The checklist manifesto : how to get things right. 2010, New York, N.Y., London: Metropolitan Books, Profile Books.
3. Birkmeyer, J.D., Strategies for improving surgical quality--checklists and beyond. *N Engl J Med* 2010;363(20):1963-5.
4. Hearns, S., Checklists in emergency medicine. *Emergency Medicine Journal* 2018;35(9):530-531.
5. Goldhaber-Fiebert, S.N., S. Bereknyei Merrell, A.V. Agarwala, et al., Clinical Uses and Impacts of Emergency Manuals During Perioperative Crises. *Anesth Analg* 2020;131(6):1815-1826.
6. Burian, B.K., A. Clebone, K. Dismukes, et al., More Than a Tick Box: Medical Checklist Development, Design, and Use. *Anesth Analg* 2018;126(1):223-232.
7. Clebone, A., B.K. Burian, and A. Tung, Matching design to use: a task analysis comparison of three cognitive aid designs used during simulated crisis management. *Can J Anaesth* 2019;66(6):658-671.
8. Verdaasdonk, E.G., L.P. Stassen, P.P. Widhiasmara, et al., Requirements for the design and implementation of checklists for surgical processes. *Surg Endosc* 2009;23(4):715-26.

For peer review only - http://bmjopen.bmj.com/site/about/guidelines.xhtml

9. Haynes, A.B., T.G. Weiser, W.R. Berry, et al., A surgical safety checklist to reduce morbidity and mortality in a global population. *N Engl J Med* 2009;360(5):491-9.
10. de Vries, E.N., H.A. Prins, R.M. Crolla, et al., Effect of a comprehensive surgical safety system on patient outcomes. *N Engl J Med* 2010;363(20):1928-37.
11. Sotto, K.T., B.K. Burian, and M. Brindle, Impact of the WHO Surgical Safety Checklist Relative to Its Design and Intended Use: A Systematic Review and Meta-Meta-Analysis. *J Am Coll Surg* 2021.
12. Pronovost, P., D. Needham, S. Berenholtz, et al., An intervention to decrease catheter-related bloodstream infections in the ICU. *N Engl J Med* 2006;355(26):2725-32.
13. Pronovost, P.J., C.A. Goeschel, E. Colantuoni, et al., Sustaining reductions in catheter related bloodstream infections in Michigan intensive care units: observational study. *BMJ* 2010;340:c309.
14. Bion, J., A. Richardson, P. Hibbert, et al., 'Matching Michigan': a 2-year stepped interventional programme to minimise central venous catheter-blood stream infections in intensive care units in England. *BMJ Qual Saf* 2013;22(2):110-23.
15. LeBlanc, V.R., The effects of acute stress on performance: implications for health professions education. *Acad Med* 2009;84(10 Suppl):S25-33.
16. Dismukes, R., T.E. Goldsmith, and J.A. Kochan, *Effects of acute stress on aircrew performance: literature review and analysis of operational aspects (NASA/TM-2015-218930)*. 2015, National Aeronautics and Space Administration: Moffett Field, CA.
17. Arriaga, A.F., A.M. Bader, J.M. Wong, et al., Simulation-based trial of surgical-crisis checklists. *N Engl J Med* 2013;368(3):246-53.
18. Just, K.S., S. Hubrich, D. Schmidtke, et al., The effectiveness of an intensive care quick reference checklist manual--a randomized simulation-based trial. *J Crit Care* 2015;30(2):255-60.
19. Koers, L., M. van Haperen, C.G.F. Meijer, et al., Effect of Cognitive Aids on Adherence to Best Practice in the Treatment of Deteriorating Surgical Patients: A Randomized Clinical Trial in a Simulation Setting. *JAMA Surg* 2020;155(1):e194704.
20. Hall, C., D. Robertson, M. Rolfe, et al., Do cognitive aids reduce error rates in resuscitation team performance? Trial of emergency medicine protocols in simulation training (TEMPIST) in Australia. *Hum Resour Health* 2020;18(1):1.
21. Knoche, B.B., C. Busche, M. Grodd, et al., A simulation-based pilot study of crisis checklists in the emergency department. *Intern Emerg Med* 2021.
22. Dryver, E., J. Lundager Forberg, C. Hård Af Segerstad, et al., Medical crisis checklists in the emergency department: a simulation-based multi-institutional randomised controlled trial. *BMJ Qual Saf* 2021;30(9):697-705.
23. Simmons, W.R. and J. Huang, Operating Room Emergency Manuals Improve Patient Safety: A Systemic Review. *Cureus* 2019;11(6):e4888.
24. Ranganathan, P., J.H. Phillips, A.F. Attaallah, et al., The use of cognitive aid checklist leading to successful treatment of malignant hyperthermia in an infant undergoing cranioplasty. *Anesth Analg* 2014;118(6):1387.
25. Bereknyei Merrell, S., D.M. Gaba, A.V. Agarwala, et al., Use of an Emergency Manual During an Intraoperative Cardiac Arrest by an Interprofessional Team: A Positive-Exemplar Case Study of a New Patient Safety Tool. *Jt Comm J Qual Patient Saf* 2018;44(8):477-484.
26. Huang, J., F. Jiang, J.-F. Zhang, et al., The use of emergency manuals leading to successful treatment of severe bronchospasm. *The Journal of Medical Practice Management: MPM* 2018;33(5):330-332.
27. Carayon, P., T.B. Wetterneck, A.J. Rivera-Rodriguez, et al., Human factors systems approach to healthcare quality and patient safety. *Appl Ergon* 2014;45(1):14-25.

For peer review only - http://bmjopen.bmj.com/site/about/guidelines.xhtml

28. Luedi, M.M. and M.B. Weinger, Emergency Manuals in Context: One Component of Resilient Performance. *Anesthesia & Analgesia* 2020;131(6):1812-1814.
29. Kapadia, M.R. and S.J. Ivatury, Transitioning Cognitive Aids for Training to Tools for Rescue in Clinical Practice. *JAMA Surgery* 2020;155(1):e194705-e194705.
30. Goldhaber-Fiebert, S.N. and C. Macrae, Emergency Manuals: How Quality Improvement and Implementation Science Can Enable Better Perioperative Management During Crises. *Anesthesiol Clin* 2018;36(1):45-62.
31. Burden, A.R., Z.J. Carr, G.W. Staman, et al., Does every code need a "reader?" improvement of rare event management with a cognitive aid "reader" during a simulated emergency: a pilot study. *Simul Healthc* 2012;7(1):1-9.
32. World Health Organization & WHO Patient Safety, *Implementation Manual - WHO Surgical Safety Checklist (First Edition)*. 2008, World Health Organization: https://apps.who.int/iris/handle/10665/70046.
33. Härtel, C., G. Prosen, R. Brown, et al., *European Core Curriculum for Emergency Medicine. Version 2.0*. 2019 [cited 27 july 2019], European Society for Emergency Medicine: https://eusem.org/images/Curriculum_2.0_WEB.pdf.
34. Civil Aviation Authority (UK) *CAP 708: Guidance on the Design, Presentation and Use of Electronic Checklists*. 2005.
35. Civil Aviation Authority (UK) *CAP 676: Guidance on the Design, Presentation and Use of Emergency and Abnormal Checklists*. 2006.
36. Burian, B.K., Design Guidance for Emergency and Abnormal Checklists in Aviation. *Proceedings of the Human Factors and Ergonomics Society Annual Meeting* 2006;50(1):106-110.
37. Hales, B., M. Terblanche, R. Fowler, et al., Development of medical checklists for improved quality of patient care. *Int J Qual Health Care* 2008;20(1):22-30.
38. Hepner, D.L., A.F. Arriaga, J.B. Cooper, et al., Operating Room Crisis Checklists and Emergency Manuals. *Anesthesiology* 2017;127(2):384-392.
39. Clebone, A., B.K. Burian, and A. Tung, The effect of cognitive aid design on the perceived usability of critical event cognitive aids. *Acta Anaesthesiol Scand* 2020;64(3):378-384.
40. Dryver, E., Emergency Medicine Checklist Compendium. 2022: Media-Tryck, Lund University.
41. Borshoff, D. and S. Weingart, The Resuscitation Crisis Manual. 2018: Leeuwin Press.
42. Goldhaber-Fiebert, S.N., N. Austin, E. Sultan, et al., *Emergency Manual: Cognitive aids for perioperative crises*. 2021: http://emergencymanual.stanford.edu
43. Robertson, D., *Emergency Protocols*, C. Hall, J. Mackenzie, and L. Murray, Editors. 2021: http://emergencyprotocols.org.au.
44. Borshoff, D., The limitations of crisis checklists. *Anesth Analg* 2014;118(6):1387-8.
45. Sollid, S.J.M. and A. Kämäräinen, The checklist, your friend or foe? *Acta Anaesthesiol Scand* 2020;64(1):4-5.

For peer review only

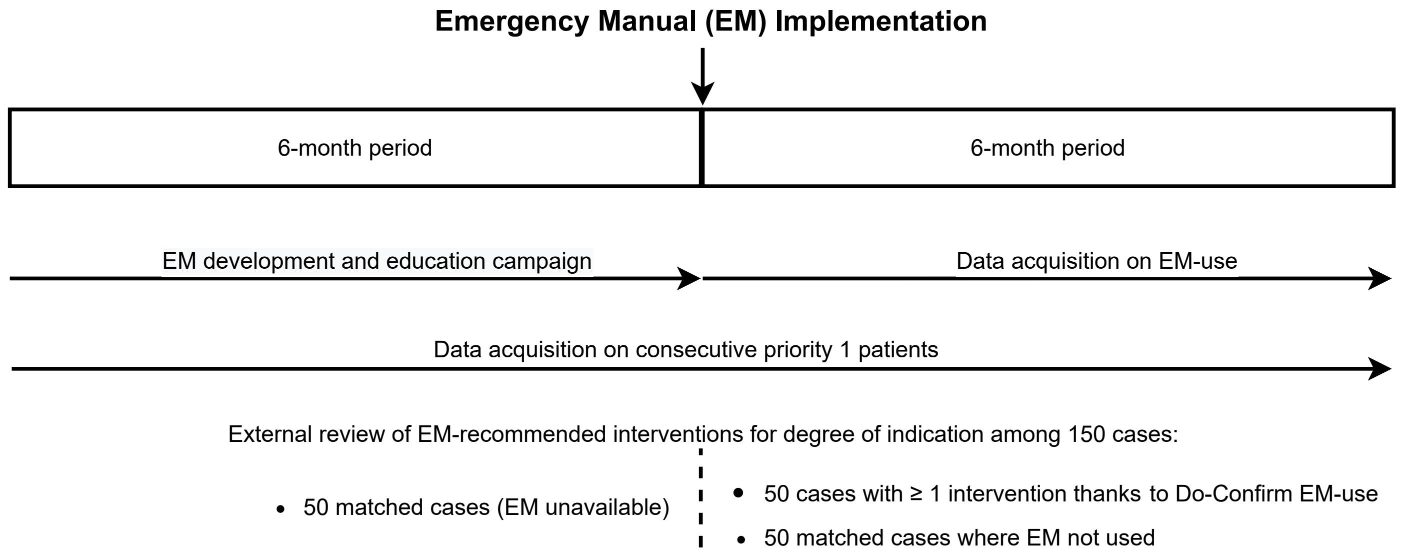
Emergency Manual (EM) Implementation
6-month period
6-month period
EM development and education campaign
Data acquisition on EM-use
Data acquisition on consecutive priority 1 patients
External review of EM-recommended interventions for degree of indication among 150 cases:
50 matched cases (EM unavailable)
50 cases with ≥ 1 intervention thanks to Do-Confirm EM-use
50 matched cases where EM not used

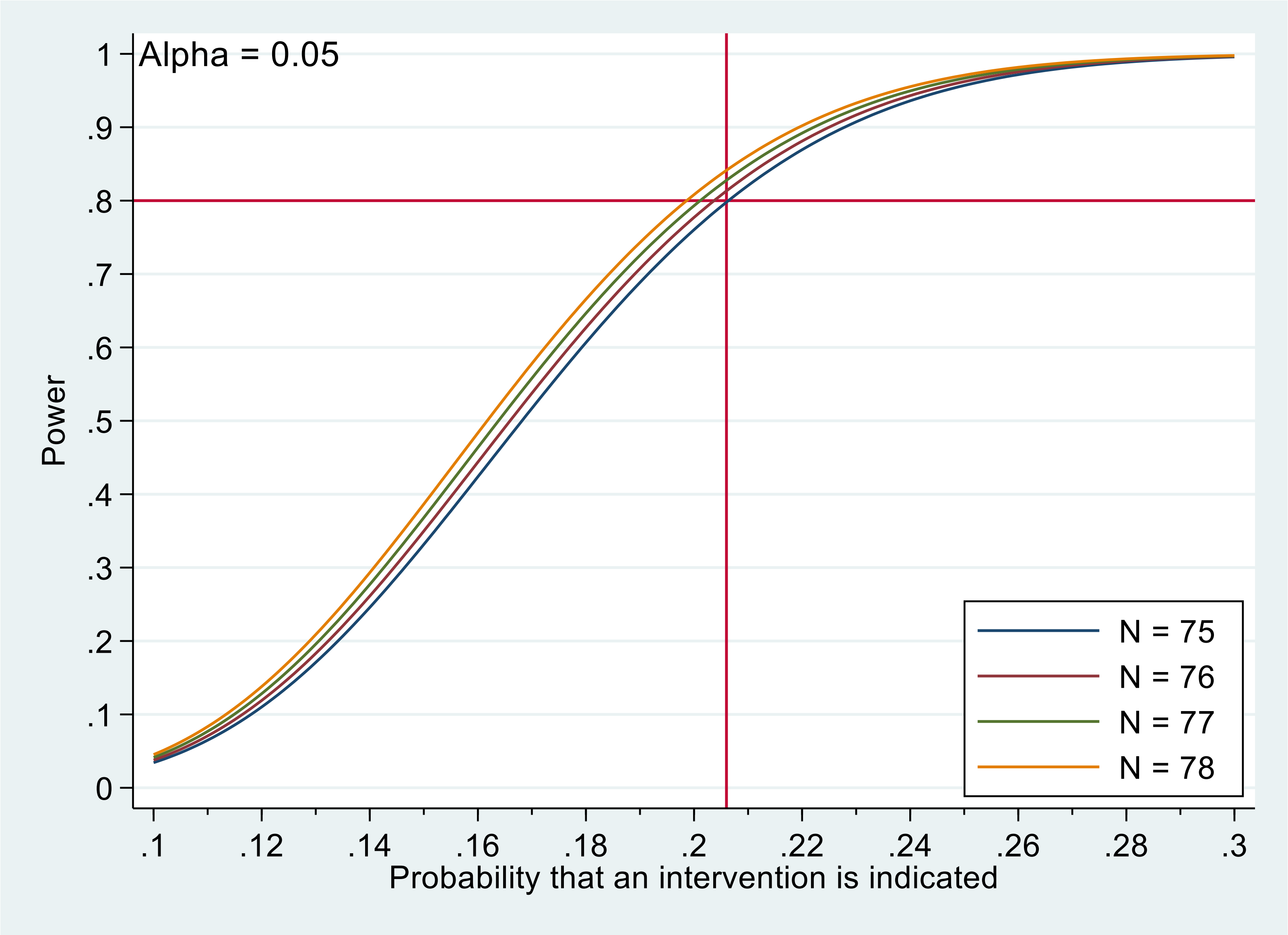
Alpha = 0.05
Power
1
.9
.8
.7
.6
.5
.4
.3
.2
.1
0
.1
.12
.14
.16
.18
.2
.22
.24
.26
.28
.3
Probability that an intervention is indicated
N = 75
N = 76
N = 77
N = 78

Appendix A: Structured Interview Guide for Reported Specific Cases

Introduction: You have requested to speak with us about the use of the Emergency Manual during the management of a specific patient in the resuscitation room. We are interested in understanding how the Emergency Manual is used, how it impacts on actual clinical care and how it can be improved.

Important information:
This project has been approved by the Swedish Ethical Review Authority
The names of specific health care personnel are not recorded.
This information obtained through this interview will not be used for any institutional process for assessing clinical management.

Background Information

Date of the interview:	
Date specific case managed:	
Interviewee's profession:	☐ Nursing assistant ☐ Nurse ☐ Resident ☐ Specialist. Physician's specialty:
Physician in charge of case	☐ Resident ☐ Specialist. Specialty:
Patient age and sex	____________ years. ☐ Male ☐ Female
Presenting complaint	
Suspected diagnosis	

Events or concerns relating to EM use (free text summary, professions of individuals recorded but names NOT recorded)

Emergency Manual Use

Sections of EM relevant to the event	
Mode of EM use	☐ Do-Confirm ☐ Read-Do ☐ Sampling

For peer review only

Assessed impact of EM use on patient care (clinical decisions regarding diagnosis, treatment; free text summary)

Summary of Impact of EM use on patient care: ☐ Negative ☐ Positive

Assessed impact of EM use on team members/teamwork (organization, communication; free text summary)

Summary of Impact of EM use on team members/teamwork: ☐ Negative ☐ Positive

Verbatim summary of how the impact of EM access on the specific case:

Suggestions for improvement (free text summary):

For peer review only

Reporting checklist for protocol of a clinical trial.

Based on the SPIRIT guidelines.

Instructions to authors

Complete this checklist by entering the page numbers from your manuscript where readers will find each of the items listed below.

Your article may not currently address all the items on the checklist. Please modify your text to include the missing information. If you are certain that an item does not apply, please write "n/a" and provide a short explanation.

Upload your completed checklist as an extra file when you submit to a journal.

In your methods section, say that you used the SPIRITreporting guidelines, and cite them as:

Chan A-W, Tetzlaff JM, Gøtzsche PC, Altman DG, Mann H, Berlin J, Dickersin K, Hróbjartsson A, Schulz KF, Parulekar WR, Krleža-Jerić K, Laupacis A, Moher D. SPIRIT 2013 Explanation and Elaboration: Guidance for protocols of clinical trials. BMJ. 2013;346:e7586

		Reporting Item	Page Number
**Administrative information**			
Title	#1	Descriptive title identifying the study design, population, interventions, and, if applicable, trial acronym	1
Trial registration	#2a	Trial identifier and registry name. If not yet registered, name of intended registry	3
Trial registration: data set	#2b	All items from the World Health Organization Trial Registration Data Set	n/a (used registry: clinicaltrials.gov)
Protocol version	#3	Date and version identifier	n/a (version 1)
Funding	#4	Sources and types of financial, material, and other support	20
Roles and responsibilities: contributorship	#5a	Names, affiliations, and roles of protocol contributors	1

For peer review only

Roles and responsibilities: sponsor contact information	#5b	Name and contact information for the trial sponsor	1
Roles and responsibilities: sponsor and funder	#5c	Role of study sponsor and funders, if any, in study design; collection, management, analysis, and interpretation of data; writing of the report; and the decision to submit the report for publication, including whether they will have ultimate authority over any of these activities	20
Roles and responsibilities: committees	#5d	Composition, roles, and responsibilities of the coordinating centre, steering committee, endpoint adjudication committee, data management team, and other individuals or groups overseeing the trial, if applicable (see Item 21a for data monitoring committee)	n/a
**Introduction**			
Background and rationale	#6a	Description of research question and justification for undertaking the trial, including summary of relevant studies (published and unpublished) examining benefits and harms for each intervention	5
Background and rationale: choice of comparators	#6b	Explanation for choice of comparators	n/a (the comparison is management with vs without access to an EM)
Objectives	#7	Specific objectives or hypotheses	8
Trial design	#8	Description of trial design including type of trial (eg, parallel group, crossover, factorial, single group), allocation ratio, and framework (eg, superiority, equivalence, non-inferiority, exploratory)	9
**Methods: Participants,**			

For peer review only

**interventions, and outcomes**			
Study setting	#9	Description of study settings (eg, community clinic, academic hospital) and list of countries where data will be collected. Reference to where list of study sites can be obtained	9
Eligibility criteria	#10	Inclusion and exclusion criteria for participants. If applicable, eligibility criteria for study centres and individuals who will perform the interventions (eg, surgeons, psychotherapists)	9
Interventions: description	#11a	Interventions for each group with sufficient detail to allow replication, including how and when they will be administered	10
Interventions: modifications	#11b	Criteria for discontinuing or modifying allocated interventions for a given trial participant (eg, drug dose change in response to harms, participant request, or improving / worsening disease)	15
Interventions: adherance	#11c	Strategies to improve adherence to intervention protocols, and any procedures for monitoring adherence (eg, drug tablet return; laboratory tests)	11
Interventions: concomitant care	#11d	Relevant concomitant care and interventions that are permitted or prohibited during the trial	11
Outcomes	#12	Primary, secondary, and other outcomes, including the specific measurement variable (eg, systolic blood pressure), analysis metric (eg, change from baseline, final value, time to event), method of aggregation (eg, median, proportion), and time point for each outcome. Explanation of the clinical relevance of chosen efficacy and harm outcomes is strongly recommended	13

For peer review only

Participant timeline	#13	Time schedule of enrolment, interventions (including any run-ins and washouts), assessments, and visits for participants. A schematic diagram is highly recommended (see Figure)	9 + Figure 1
Sample size	#14	Estimated number of participants needed to achieve study objectives and how it was determined, including clinical and statistical assumptions supporting any sample size calculations	14 + Figure 2
Recruitment	#15	Strategies for achieving adequate participant enrolment to reach target sample size	n/a (consecutive priority 1 patients treated in the ED)
**Methods: Assignment of interventions (for controlled trials)**			
Allocation: sequence generation	#16a	Method of generating the allocation sequence (eg, computer-generated random numbers), and list of any factors for stratification. To reduce predictability of a random sequence, details of any planned restriction (eg, blocking) should be provided in a separate document that is unavailable to those who enrol participants or assign interventions	n/a (one arm study)
Allocation concealment mechanism	#16b	Mechanism of implementing the allocation sequence (eg, central telephone; sequentially numbered, opaque, sealed envelopes), describing any steps to conceal the sequence until interventions are assigned	n/a
Allocation: implementation	#16c	Who will generate the allocation sequence, who will enrol participants, and who will assign participants to interventions	n/a

For peer review only

Blinding (masking)	#17a	Who will be blinded after assignment to interventions (eg, trial participants, care providers, outcome assessors, data analysts), and how	14
Blinding (masking): emergency unblinding	#17b	If blinded, circumstances under which unblinding is permissible, and procedure for revealing a participant's allocated intervention during the trial	n/a (no blinding for intervention, i.e. access to checklists)
**Methods: Data collection, management, and analysis**			
Data collection plan	#18a	Plans for assessment and collection of outcome, baseline, and other trial data, including any related processes to promote data quality (eg, duplicate measurements, training of assessors) and a description of study instruments (eg, questionnaires, laboratory tests) along with their reliability and validity, if known. Reference to where data collection forms can be found, if not in the protocol	12
Data collection plan: retention	#18b	Plans to promote participant retention and complete follow-up, including list of any outcome data to be collected for participants who discontinue or deviate from intervention protocols	n/a (consecutive patients management in the ED)
Data management	#19	Plans for data entry, coding, security, and storage, including any related processes to promote data quality (eg, double data entry; range checks for data values). Reference to where details of data management procedures can be found, if not in the protocol	12
Statistics: outcomes	#20a	Statistical methods for analysing primary and secondary outcomes. Reference to	16

For peer review only

		where other details of the statistical analysis plan can be found, if not in the protocol	
Statistics: additional analyses	#20b	Methods for any additional analyses (eg, subgroup and adjusted analyses)	16
Statistics: analysis population and missing data	#20c	Definition of analysis population relating to protocol non-adherence (eg, as randomised analysis), and any statistical methods to handle missing data (eg, multiple imputation)	n/a
**Methods: Monitoring**			
Data monitoring: formal committee	#21a	Composition of data monitoring committee (DMC); summary of its role and reporting structure; statement of whether it is independent from the sponsor and competing interests; and reference to where further details about its charter can be found, if not in the protocol. Alternatively, an explanation of why a DMC is not needed	n/a (checklist use is not mandatory; there is no evidence that checklist access is hazardous to patient management; teams can request immediate contact with the investigators to report specific events associated with checklist use)
Data monitoring: interim analysis	#21b	Description of any interim analyses and stopping guidelines, including who will have access to these interim results and make the final decision to terminate the trial	n/a
Harms	#22	Plans for collecting, assessing, reporting, and managing solicited and spontaneously reported adverse events and other unintended effects of trial interventions or trial conduct	15
Auditing	#23	Frequency and procedures for auditing trial conduct, if any, and whether the process will be independent from investigators and the sponsor	n/a (this study focuses on how teams use a new tool)
**Ethics and dissemination**			
Research ethics	#24	Plans for seeking research ethics committee	18

For peer review only

approval		/ institutional review board (REC / IRB) approval	
Protocol amendments	#25	Plans for communicating important protocol modifications (eg, changes to eligibility criteria, outcomes, analyses) to relevant parties (eg, investigators, REC / IRBs, trial participants, trial registries, journals, regulators)	n/a (consecutive unselected patients managed in the emergency room)
Consent or assent	#26a	Who will obtain informed consent or assent from potential trial participants or authorised surrogates, and how (see Item 32)	17
Consent or assent: ancillary studies	#26b	Additional consent provisions for collection and use of participant data and biological specimens in ancillary studies, if applicable	n/a (no such data)
Confidentiality	#27	How personal information about potential and enrolled participants will be collected, shared, and maintained in order to protect confidentiality before, during, and after the trial	12
Declaration of interests	#28	Financial and other competing interests for principal investigators for the overall trial and each study site	20
Data access	#29	Statement of who will have access to the final trial dataset, and disclosure of contractual agreements that limit such access for investigators	13
Ancillary and post trial care	#30	Provisions, if any, for ancillary and post-trial care, and for compensation to those who suffer harm from trial participation	n/a
Dissemination policy: trial results	#31a	Plans for investigators and sponsor to communicate trial results to participants, healthcare professionals, the public, and other relevant groups (eg, via publication, reporting in results databases, or other data	18

For peer review only

		sharing arrangements), including any publication restrictions	
Dissemination policy: authorship	#31b	Authorship eligibility guidelines and any intended use of professional writers	n/a
Dissemination policy: reproducible research	#31c	Plans, if any, for granting public access to the full protocol, participant-level dataset, and statistical code	n/a
**Appendices**			
Informed consent materials	#32	Model consent form and other related documentation given to participants and authorised surrogates	n/a (see above)
Biological specimens	#33	Plans for collection, laboratory evaluation, and storage of biological specimens for genetic or molecular analysis in the current trial and for future use in ancillary studies, if applicable	n/a (no such data collected)

None The SPIRIT Explanation and Elaboration paper is distributed under the terms of the Creative Commons Attribution License CC-BY-NC. This checklist can be completed online using https://www.goodreports.org/, a tool made by the EQUATOR Network in collaboration with Penelope.ai

For peer review only

BMJ Open

Clinical use of an emergency manual by resuscitation teams and impact on performance in the emergency department—a prospective mixed-methods study protocol

Journal:	*BMJ Open*
Manuscript ID	bmjopen-2022-071545.R2
Article Type:	Protocol
Date Submitted by the Author:	01-Jul-2023
Complete List of Authors:	Dryver, Eric; Skåne University Hospital Lund, Department of Emergency and Internal Medicine; Lund University, Department of Clinical Sciences at Lund<br>Olsson de Capretz, Pontus; Skåne University Hospital Lund, Department of Emergency and Internal Medicine; Lund University, Department of Clinical Sciences at Lund<br>Mohammad, Mohammed; Skåne University Hospital Lund, Department of Emergency and Internal Medicine<br>Armelin, Malin; Skåne University Hospital Lund, Department of Emergency and Internal Medicine<br>Dupont, William D. ; Vanderbilt University Medical Center, Department of Biostatistics<br>Bergenfelz, Anders; Lund University, Department of Clinical Sciences at Lund<br>Ekelund, Ulf; Skåne University Hospital Lund, Department of Emergency and Internal Medicine; Lund University, Department of Clinical Sciences at Lund
**Primary Subject Heading**:	Emergency medicine
Secondary Subject Heading:	Emergency medicine
Keywords:	ACCIDENT & EMERGENCY MEDICINE, INTENSIVE & CRITICAL CARE, Protocols & guidelines < HEALTH SERVICES ADMINISTRATION & MANAGEMENT

SCHOLARONE™ Manuscripts

TITLE PAGE

Clinical use of an emergency manual by resuscitation teams and impact on performance in the emergency department—a prospective mixed-methods study protocol

AUTHORS

Eric Dryver[*†‡], M.D. eric.dryver@med.lu.se
Pontus Olsson de Capretz[*†], M.D. pontus.olsson_de_capretz@med.lu.se
Mohammed Mohammad[*], M.D. mohammed.mohammad@skane.se
Malin Armelin[*], RN, MSN Malin.Armelin@skane.se
William D Dupont[§], Ph.D. william.dupont@vumc.org
Anders Bergenfelz[†], M.D., Ph.D. anders.bergenfelz@med.lu.se
Ulf Ekelund[*†], M.D., Ph.D. ulf.ekelund@med.lu.se

* Department of Emergency and Internal Medicine, Skåne University Hospital at Lund, Sweden
† Department of Clinical Sciences at Lund, Lund University, Sweden
‡ Practicum Clinical Skills Centre, Lund, Sweden
§ Department of Biostatistics, Vanderbilt University Medical Center, USA

CORRESPONDING AUTHOR

Eric Dryver
Department of Emergency Medicine
Skåne University Hospital at Lund
Klinikgatan 15
22185 Lund
Sweden
eric.dryver@med.lu.se

WORD COUNT

4227

For peer review only

ABSTRACT

Introduction

Simulation-based studies indicate that crisis checklist use improves management of patients with critical conditions in the emergency department (ED). An interview-based study suggests that use of an emergency manual (EM)—a collection of crisis checklists—improves management of clinical perioperative crises. There is a need for in-depth prospective studies of EM use during clinical practice, evaluating when and how EMs are used and impact on patient management.

Methods and analysis

This six-month-long study prospectively evaluates a digital EM during management of priority 1 patients in the Skåne University Hospital at Lund's ED. Resuscitation teams are encouraged to use the EM after a management plan has been derived ("Do-Confirm"). The documenting nurse activates and reads from the EM, and checklists are displayed on a large screen visible to all team-members. Whether the EM is activated, and which sections are displayed, are automatically recorded. Interventions performed thanks to Do-Confirm EM use are registered by the nurse. Fifty cases featuring such interventions are reviewed by specialists in emergency medicine blinded to whether the interventions were performed prior to or after EM use. All interventions are graded as indicated, of neutral relevance or not indicated. The primary outcome measures are the proportions of interventions performed thanks to Do-Confirm EM use graded as indicated, of neutral relevance, and not indicated. A secondary outcome measure is the team's subjective evaluation of the EM's value on a Likert scale of 1 to 6. Team members can report events related to EM use, and information from these events are extracted through structured interviews.

For peer review only - http://bmjopen.bmj.com/site/about/guidelines.xhtml

Ethics and dissemination

The study is approved by the Swedish Ethical Review Authority (Dnr 2022-01896-01). Results will be published in a peer-reviewed journal and abstracts submitted to national and international conferences to disseminate our findings.

Registration

The trial is registered at clinicaltrials.gov (NCT 05649891).

For peer review only

For peer review only - http://bmjopen.bmj.com/site/about/guidelines.xhtml

STRENGTHS AND LIMITATIONS OF THIS STUDY

- To date, the majority of studies of Emergency Manuals (EM)—collections of crisis checklists— have been simulation-based, with the notable exceptions of one interview-based and one prospective study. This study prospectively evaluates an EM during the management of unselected, consecutive priority 1 patients in an emergency department over a six-month period.
- Some of the data on patient characteristics and EM use are automatically registered, while other EM use data are actively registered by the documenting nurse.
- The study aims to determine whether interventions carried out thanks to checklist use are indicated, of neutral relevance or not indicated based on the clinical context, as assessed by external reviewers.
- The external review process is based on information available from patient records, which may not convey all the relevant factors affecting clinical decisions.
- The study is not a randomized-controlled trial and is not powered nor designed to detect causal relationship between EM use and patient outcome.

For peer review only

INTRODUCTION

Checklists

A checklist is a cognitive aid—consisting of a list of assessments or actions—designed to be carried out systematically. The function of checklists is to improve task performance; checklists are essentially knowledge-translation tools that promote completeness, consistency and alignment with best practice guidelines when carrying out a task [1]. Checklists work by directing focus, providing facts [2], and guiding—though not dictating—decision-making. Checklists are also believed to improve performance through other mechanisms, such as reducing mental workload, promoting a shared mental model among team-members and empowering team-members to voice concerns [3-5].

There are two basic modes of checklist use [6]. In Read-Do mode, checklists are used as the task is performed to guide task completion. During the Do-Confirm mode, checklists are used after task performance to verify completeness. Cognitive aids may even be used to retrieve specific information (for instance the dosage of a specific medication in a specific setting), so-called Sampling mode [6, 7]. Checklists may be used by one or several people, silently or read aloud [6], and can be categorized according to the circumstances in which they are used: normal (or routine) situations, emergency situations, and atypical (or non-normal) situations [6, 8].

Checklists for routine situations in health care

The World Health Organization (WHO) Surgical Safety Checklist [9] and the Surgical Safety Patient System [10] are examples of checklists used to improve performance during routine surgical procedures. There is substantial, robust, international evidence that use of the WHO Surgical Safety Checklist reduces mortality and complications associated with general

For peer review only - http://bmjopen.bmj.com/site/about/guidelines.xhtml

surgery [11]. A checklist designed to minimize bloodstream infections associated with the routine insertion of central venous catheters in the intensive care unit is also associated with improved performance during routine health care delivery [12-14].

Checklists for health care emergencies

Stress affects attention and information retrieval from memory [15, 16], and hence checklists may play a particular role in improving performance during stressful emergencies [1, 4]. Due to the unexpected, low-frequency nature of medical emergencies, crisis checklists have mainly been studied using simulation. One study reported a reduction in the frequency of unperformed lifesaving processes of care from 23% to 6% associated with checklist access during simulated surgical crises [17]. Another study reported that checklist use increased the number of key items performed during scenarios in a simulated intensive care unit from 7 to 9 [18]. An additional study performed in a simulated surgical ward reported that use of a cognitive aid reduced the frequency of omitted critical management steps from 33% to 10% [19].

Two studies evaluated crisis checklists in a simulated resuscitation room. One study evaluated the use of an Emergency Protocols Handbook through four scenarios and reported that checklist use decreased error rate from 39% to 19% [20]. The other study evaluated a collection of four checklists through four scenarios; checklist use was associated with an increased frequency of hemodynamic stabilization and diagnosis recognition, as well as an increased adherence to critical steps (56% without checklist, 82% with checklists) [21].

A simulation-based, randomized controlled trial of medical crisis checklists was performed in four resuscitation rooms using actual resuscitation teams during their clinical shifts [22].

For peer review only - http://bmjopen.bmj.com/site/about/guidelines.xhtml

Eight medical crises were simulated. With checklist access, the median percentage of key interventions performed was 86%; without access, the median percentage was 39%.

Evidence that collections of crisis checklists and cognitive aid elements such as medication doses—Emergency Manuals (EM)—improve actual clinical care is limited. Several reports document successful management of perioperative crises thanks to the use of operating room EMs [23-26]. An interview-based study of anaesthesiologists involved in perioperative crises in 69 patients revealed that EM use led to identifying and addressing errors of omission in 59% of cases, while lack of EM use was associated with self-identified errors of omission or delays in intervention performance in 56% of cases [5]. There is also a lack of prospective studies of EM use. One recently published prospective study reported sustained use in the operative setting multiple years after initial implementation [27].

Systematic crisis checklist evaluation in the clinical setting

Performance is the end-product of complex interactions between people, the tools at their disposal, and the physical and cultural environments they work in [28]. Checklists do not work in isolation, and their impact on performance is contingent not only upon their content and design but also on when, where, how and by whom they are used. Simulation-based trials and interview-based studies [29] provide limited evidence for how checklists may actually impact resuscitation team performance in a specific clinical setting. Several authors have called for pilot-studies that systematically evaluate crisis checklists in the clinical setting [5, 19, 20, 22, 29, 30].

Experts emphasize the importance of establishing the rationale for EM use, fostering a sense of local EM ownership, ensuring access and familiarity with the tool, and customization [31].

For peer review only

In particular, an explicit protocol is likely required to ensure that checklists are used as intended. A simulation-based study reported that the mere access to crisis checklists did not ensure their systematic use; only when an assigned team-member read aloud the steps on the checklist were all critical actions pe rformed [32]. The Surgical Safety Checklist Implementation Manual emphasizes the importance of clearly assigning the role of checklist implementation to a specific person[33] and an interview-based study of EM use during perioperative crises reported beneficial effects of a reader-leader combination [5].

Challenges to implementation and evaluation of an EM in the ED include the following:

- the number of possible presenting complaints, acute conditions and pre-existing co-morbidities among priority 1 patients is large, and the degree of actual acuity is variable
- the number of personnel that work in the resuscitation room is large, and each personnel has variable context-specific degrees of expertise; managing a patient with acute pulmonary edema may be "routine" for a seasoned physician and an "emergency" for a junior resident
- Do-Confirm mode of checklist use is best suited to quantify the added value of checklist use, while Read-Do and Sampling modes may be best suited for checklist use during an actual crisis
- Ensuring that checklists relevant to the clinical context are made available to all team members at a suitable juncture during patient management, and encouraging checklist use without negatively impacting on team dynamics, are not self-evident tasks

These challenges have informed the study design.

Objectives

For peer review only - http://bmjopen.bmj.com/site/about/guidelines.xhtml

The primary objective of the study is to determine whether Do-Confirm EM use leads to the performance, by resuscitation teams managing priority 1 patients in the ED, of additional interventions that are deemed indicated, of neutral relevance or not indicated given the clinical context. Secondary study objectives are

- determining the frequency and modality (Do-Confirm, Read-Do, Sampling) with which the EM is used and whether these changed significantly over the study period
- determining whether Do-Confirm EM use in selected cases where other modes were used might have led to the performance of additional indicated interventions
- determining the degree to which resuscitation teams find the EM helpful, and under which circumstances

METHODS AND ANALYSIS

Setting, patient population and personnel

The study will be carried out in the resuscitation room of the ED of Skåne's University Hospital at Lund (referred to hereafter as Lund's RR). Lund's RR is designed for the simultaneous management of three priority 1 patients. EM access during clinical practice will be provided in September 2023. The study population consists of consecutive priority 1 patients managed in the Lund's RR during the six-month period prior to, and the six-month pilot study period following EM implementation. Patients are deemed to be priority 1 based on vital signs and symptoms according to the Rapid Emergency Triage and Treatment System (http://predicare.se/) the most commonly used triage system in Sweden. The yearly number of priority 1 patients treated in Lund's RR is roughly 4000 and consists of adult patients (> 18 years) with medical or surgical emergencies and patients of all ages with level 1 trauma. Most of these patients are brought to the RR via ambulance, and only occasionally are they identified at the triage or at later stage of ED care. All priority 1 patients are

For peer review only - http://bmjopen.bmj.com/site/about/guidelines.xhtml

managed immediately in the RR by multidisciplinary resuscitation teams. The core resuscitation team consists of a resident or specialist in emergency medicine, two nurses and one nursing assistant. When the patient is especially critical (for instance cardiac arrest, level 1 trauma, upper airway obstruction), additional personnel (anaesthesiologists, surgeons, cardiologists, otorhinolaryngologists) supplement the resuscitation team.

Emergency Manual: content and format

The Emergency Manual consists of problem checklists, diagnosis checklists, procedure checklists and fact sheets in digital form. The checklists and fact sheets are accessed through a tablet computer located at each of the three resuscitation sections in the RR and displayed on a large screen visible to all members of the resuscitation team. Problem checklists consist of lists of interventions and diagnoses to consider based on the patient's presenting symptoms. The popover windows display key clinical features suggestive of specific diagnoses. Diagnosis checklists consist of lists of interventions to consider during the management of a suspected diagnosis. Each intervention is associated with a popover window displaying indications, contraindications or risks associated with the intervention, and details regarding how the intervention is performed, as described in a previous study [22]. Procedure checklists provide step-by-step guidance for the performance of key procedures as well as pictures. Fact sheets provide information such as medical dosages for sedation and anaesthesia for patients of all ages.

The collection of checklists and fact sheets covers the majority of symptoms, diagnoses and situations that may present in the RR, in accordance with the current European Core Curriculum in Emergency Medicine [34]. The format and design of each checklist or fact sheet is informed by the literature pertaining to emergency and abnormal checklists in the

For peer review only - http://bmjopen.bmj.com/site/about/guidelines.xhtml

aviation industry [35-37], articles on medical checklists [6, 38-40] and a selection of EMs [41-44]. The content is customized to local circumstances in Lund's ED (available medications, medication locations, local routines and phone numbers). EM content and format are further developed through iterative, rapid-cycle simulation-based testing and review by specialists, residents, nurses and nursing assistants who work clinically in Lund's RR. Internet access to the EM is provided to the ED personnel prior to and during the study to allow for familiarization and suggestions for improvement. Each checklist and fact sheet is approved by at least one specialist in emergency medicine and one nurse, both of whom work clinically in Lund's RR.

Emergency Manual: protocol for use

EM use during priority 1 management is strongly advocated but not mandatory. The documenting nurse is assigned the specific task of activating the digital EM. Regarding problem and diagnosis checklists, Do-Confirm is the default mode of EM use—the team first manages priority 1 patients without support from the EM. Once the management plan is finalized (a juncture which may be verbally confirmed with the team-leader), the documenting nurse then asks the team: "Which checklist should I display?" When a checklist is displayed, the documenting nurse reads aloud each potential intervention or diagnosis for team consideration. The documenting nurse registers on the digital checklist whether each intervention is:

- deemed indicated and has already been carried out
- deemed irrelevant or contraindicated
- deemed indicated and will be carried out thanks to EM use

The physician responsible for patient management has the mandate to request that the EM not be used, to pause or terminate EM use at any time, and to depart from the recommendations

For peer review only - http://bmjopen.bmj.com/site/about/guidelines.xhtml

featuring on the checklist. Physicians may also request that the EM be displayed directly and used in Read-Do mode or for retrieval of specific information (Sampling mode). Regarding procedure checklists and fact sheets, the default modes of use are Read-Do and Sampling respectively. After EM use, teams grade the usefulness of the EM on a 1-6 Likert scale prior to logging out. If the documenting nurse logs in to the EM but the EM is not used, the nurse selects among potential reasons for non-use prior to logging out. Teams can also electronically request contact with the investigators to report specific events related to EM use.

Educational campaign

An educational campaign targeting all personnel who work in Lund's RR precedes the implementation of the EM in clinical practice. Evidence for the potential benefits of an EM—including results from a locally-performed crisis checklist study [22]—is presented, as well as the protocol for EM use. Documenting nurses during priority 1 patient management are taught to log in to the EM, display and navigate between checklists, open popover windows, record whether interventions were not indicated, done before or thanks to checklist access, record the team's opinion of the EM's value, request contact with the investigators, and log out. Documenting nurses carry out these functions in addition to their usual documentation tasks.

Data acquisition

The following data pertaining to each priority 1 patient managed during the six-month period prior to, and the six-month pilot study period following implementation of the EM in clinical practice, are extracted from Region Skåne's clinical databases and the national data registry SVAR (Svenska Akutvårdsregistret):

For peer review only - http://bmjopen.bmj.com/site/about/guidelines.xhtml

- patient age and sex, presenting complaint and vital signs
- presumptive diagnosis in the RR
- time spent in the RR, in the ED, and admitted to the hospital
- whether the patient was admitted to the intensive care unit, and if so duration of admission
- seven-day and thirty-day mortality

The following data regarding EM use are recorded on a dedicated computer located within the hospital's firewall each time the EM is activated during the six-month pilot study period:

- log-in and log-out times
- identification number of the nurse who logged in, level of the most senior physician who was physically present at the bedside during patient management, and patient identification number (which is immediately encrypted)
- checklists and fact sheets that were displayed
- whether interventions were performed before or thanks to checklist access when the EM was used in Do-Confirm mode, or whether the EM was used in Read-Do or Sampling mode instead
- the team's subjective evaluation of the value of the EM according to a 1-6 Likert scale
- reasons for EM non-use despite logging in
- whether prompt contact with the research team is requested

The following data is extracted from the medical records of 150 selected cases (see below):

- baseline information regarding the patient's age, sex, known medical background at the time of presentation and current medications

For peer review only - http://bmjopen.bmj.com/site/about/guidelines.xhtml

- clinical information regarding the patient's presentation to the RR: ambulance report, history that can be obtained in the RR, vital signs, physical findings, electrocardiogram, bedside blood test results and point-of-care ultrasound findings
- all interventions (investigations and treatments) performed during the patient's management in the RR
- whether the physician was a specialist or resident, and years of experience
- final hospital diagnosis pertaining to the presentation in the RR

Figure 1 summarizes data acquisition during the study period. The primary investigator alone has access to the encryption key. All investigators have access to the anonymized dataset for analysis purposes.

Impact of Emergency Manual use on resuscitation team performance

The final 50 cases where the EM was used in Do-Confirm mode and interventions were performed thanks to checklist access are identified for external review. The final cases during the six-month study period are selected to allow personnel the opportunity to integrate the new tool within their clinical practice. The degree to which each intervention is indicated is independently assessed by three specialists in emergency medicine. All three assessors are blinded to whether the intervention was performed before or after EM use. Two assessors are blinded to the content of the EM, while one is involved in the development and use of the EM. Interventions are categorized, based on the available extracted clinical information, as indicated (e.g. administering antibiotics to the septic patient), of neutral relevance (e.g. measuring liver function tests for the patient with suspected benzodiazepine overdose), or not indicated (i.e. over-investigation or -treatment, e.g. ordering computed tomography to rule out pulmonary embolism for a patient with pulmonary edema). When the three assessors are

For peer review only - http://bmjopen.bmj.com/site/about/guidelines.xhtml

not in agreement regarding the degree of indication of the intervention, resolution is reached through discussion, and based on majority opinion if no consensus can be reached. Management is deemed improved when interventions categorized as indicated are carried out thanks to EM use. EM use is deemed to not add value if additional interventions are categorized as of neutral relevance. EM use is deemed detrimental if additional interventions are categorized as not indicated.

Sample size calculation

There are no published data on EM use during patient management in the ED. We derived a sample of size of 50 patients where at least one intervention was performed thanks to Do-Confirm EM use based on the following assumptions:

- Among patients with at least one intervention performed thanks to the checklist, half of these patients will have one such intervention performed while the other half will have two. Hence, 50 such patients should generate 25 + 2×25 = 75 interventions thanks to the checklist.
- When multiple checklist-indicated interventions occur in the same patient, the probability of occurrence of these interventions are independent of each other.

In our opinion, a clinically meaningful rate for indicated interventions performed thanks to checklist access is ≥ 10%. Figure 2 shows that if the true rate of clinically indicated interventions due to the checklist is ≥ 21% then 50 patients generating 75 interventions will be sufficient to reject null hypotheses that the indicated rate is ≤ 10% with at least 80% power and a Type I error probability of $\alpha \leq 0.05$.

For peer review only - http://bmjopen.bmj.com/site/about/guidelines.xhtml

Unperformed interventions recommended by the Emergency Manual

Fifty additional patients where the EM was not used during the pilot study period, and 50 additional patients that presented during the six-month period preceding EM implementation, are also identified. These 100 patients are selected based on matching to the 50 patients where the EM was used using age, presenting complaint and relevant co-morbidities. The EM is accessed to determine whether additional interventions would have been performed, had the EM been used. All interventions—those actually performed as well as those that might have been performed had the EM been used—are then assessed for degree of indication by three external reviewers as described above. The three specialists are blinded to which interventions were actually performed. Analysing this data may suggest that the EM would have improved management had it been used, with the important caveat that many factors that impact on the suitability of interventions may not be fully conveyed in the patient's chart.

Specific case descriptions

The quantitative analyses described above are designed to measure EM added value when used in Do-Confirm mode. Yet EMs are arguably designed to be used in Read-Do or Sampling mode in the setting of a true crisis. EM use may also have unforeseen impacts on patient care and team dynamics that warrant prompt remedying. Resuscitations team personnel are encouraged to request prompt contact—via the digital EM—with the research team to report events or concerns relating to EM use during specific situations. The following information is extracted from a structured interview with the personnel regarding the report (Appendix A):

- patient age, sex, presenting complaint and suspected diagnosis
- seniority of the physician initially in charge of the case
- events or concerns relating to EM use

For peer review only - http://bmjopen.bmj.com/site/about/guidelines.xhtml

- mode of EM use (Do-Confirm, Read-Do, Sampling) and sections relevant to the case
- personnel's assessment of the impact of the EM on patient care
- personnel's assessment of impact of the EM on team members and teamwork
- personnel's suggestions for EM improvement

Data analysis

Descriptive statistics are used to analyse EM use data and determine the following (average over and trends throughout the pilot study period):

- frequency of EM use and mode of use (Do-Confirm, Read-Do, or Sampling)
- which checklists or fact sheets are most frequently accessed
- time between login to and logout from the digital EM
- subjective degree of EM value and correlation with specific checklists or fact sheets

Descriptive statistics are also used to determine whether EM use is associated with length of stay in the RR, length of hospital admission and mortality. These analyses are hypothesis-generating and not intended to infer causality. For the 150 patients that are externally reviewed, descriptive statistics are used to report the proportions of interventions deemed indicated, of neutral value or not indicated.

Patient and public involvement

Patients or the public were not involved in the design of this study, nor will they be involved in its conduct or in the reporting and dissemination of our research.

ETHICS AND DISSEMINATION

Ethical and safety considerations

For peer review only - http://bmjopen.bmj.com/site/about/guidelines.xhtml

The published literature strongly supports the claim that checklists, used as cognitive aids and not as substitutes for sound clinical judgement, improve performance. Several authors have hypothesized that checklist use may lead to distraction, delays in proper management or wrong management if the wrong checklist is used [23, 45, 46]. However, published studies have not substantiated these concerns. A simulation-based trial of crisis checklists in Lund's ED did not find any delay in the initiation of patient treatment, and all but one of 15 dangerous or inappropriate interventions were carried out by teams without checklist access [22]. One study reported that EMs caused distraction during a small proportion of perioperative crises without negatively impacting on patient care [5]. According to the study protocol, the physician in charge of patient management has the mandate to terminate EM use at any time, and no constraints are imposed on how the EM is used. For these reasons, patient risk incurred from EM access is deemed minimal.

Priority 1 patients are as a rule severely impaired by an acute critical condition and not in a state where informed consent is possible or appropriate to obtain. Written information about EM use will be available in Lund's RR and provided to patients and relatives upon request. Posters informing patients and their relatives about the on-going trial and how to request non-inclusion will be placed throughout the ED. Patient identification numbers are pseudonymized automatically and data stored on a computer within the hospital's firewall and locked in a room.

No identifying features of the personnel composing the resuscitation teams are registered during the study, aside from the identity of the personnel logging into the digital EM. Names of personnel involved in the care of specific patients are not recorded during the structured interviews. In the study that evaluated checklists in simulated critical conditions in Lund's

For peer review only - http://bmjopen.bmj.com/site/about/guidelines.xhtml

RR, over 90% of the care staff stated that they would use the checklist in real crises [22]. Specific reviews of patient management will be based on information available in the medical charts, with the understanding that these cannot convey all relevant factors affecting clinical decisions. No formal complaints or chart reviews will be triggered by the case reviews in this study. For these reasons, risks incurred to resuscitation team personnel from EM access are deemed minimal. The Swedish Ethical Review Authority has approved the study (Dnr 2022-01896-01).

Dissemination plan

Results will be published in a peer-reviewed journal and abstracts submitted to national and international conferences. The simulation-based study of crisis checklists in four EDs in the Skåne Region reported that > 90% of staff at all sites would welcome checklist access during clinical care [22]. Depending on the study results, a multicentre study using customized EMs will be considered.

STRENGTHS, LIMITATIONS & SIGNIFICANCE

The literature on medical crisis checklists argues that specific checklists, used according to clear protocols within specific contexts, improve performance. Yet there are no published data on how the introduction of an EM affects the performance of resuscitation teams during the management of actual priority 1 patients in the ED. The main strength of this study is that it evaluates EM use during the management of consecutive, unselected priority 1 patients by resuscitation teams in the ED.

In this study, some of the data on patient characteristics (such as age, chief complaint) and EM use (duration of use, checklists and fact sheets displayed) are automatically registered.

For peer review only

Other EM use data (for instance subjective team evaluation of the EM value, modality of EM use) are actively registered by the documenting nurse. The study is not a randomized controlled trial of EM use, and whether the EM is used or not is at the discretion of each resuscitation team. While these study aspects limit the evaluation of how routine EM use would impact on performance, the study will provide information about the circumstances under which resuscitation teams voluntarily use the EM and deem that it adds value to clinical practice. The study stretches over a six-month period, allowing for an analysis of how resuscitation teams integrate a new tool within their clinical practice over time.

The study is not powered nor designed to detect causal relationship between EM use and patient outcome. The study will determine whether interventions carried out thanks to checklist use are indicated, of neutral relevance or not indicated based on the clinical context, as assessed by external reviewers. A limitation of this assessment stems from the fact that the external review is based on information available from patient records, which may not convey all the relevant factors affecting clinical decisions.

This study will be conducted in a single academic Swedish ED, limiting the external validity of the results. Yet this study will provide valuable information to guide the development of a tool that has the potential to improve the management of critical patients, and pave the way to multicenter studies of EM use in the ED.

COMPETING INTERESTS STATEMENT

Non declared.

AUTHORS' CONTRIBUTIONS

For peer review only - http://bmjopen.bmj.com/site/about/guidelines.xhtml

Eric Dryver conceived of, drafted the original version of, and revised the protocol.

Pontus Olsson de Capretz reviewed and revised the protocol.

Mohammed Mohammad reviewed and revised the protocol.

Malin Armelin reviewed and revised the protocol.

William D Dupont performed the sample size calculations, reviewed and revised the protocol.

Anders Bergenfelz reviewed and revised the protocol.

Ulf Ekelund conceived of, reviewed and revised the protocol.

FUNDING STATEMENT

This work was supported by an ALF research grant (2018-0152) at Skåne University Hospital and by a grant from Region Skåne (2020-0305). This study was part of the AIR Lund (Artificially Intelligent use of Registers at Lund University) research environment and received funding from the Swedish Research Council (VR; grant no. 2019-00198). There was no industry involvement. Funding organizations had no role in the planning, design or realization of the study, collection, analysis or interpretation of data, or preparation, review, or approval of the manuscript.

For peer review only

Figure 1 Legend

This figure illustrates the two phases of the study, before and after emergency manual implementation, and how data acquisition corresponds to each phase.

Figure 2 Legend

"Intervention" refers here to interventions performed by the resuscitation teams thanks to use of the EM. Interventions are graded by external assessors as indicated, neutral or not indicated based on the clinical context. The power curves in the figure plot the power to determine, with $P \leq 0.05$, that the proportion of interventions deemed indicated exceeds 10% as a function of the true probability that the intervention is indicated. Assuming that the true proportion of interventions deemed indicated is 21%, 75 interventions are required to meet these requirements. Assuming that half of the patients feature one such intervention and the other half two, then 50 patients can be expected to generate 75 interventions.

REFERENCES

1. Goldhaber-Fiebert, S.N. and S.K. Howard, Implementing emergency manuals: can cognitive aids help translate best practices for patient care during acute events? *Anesth Analg* 2013;117(5):1149-61.
2. Gawande, A., The checklist manifesto : how to get things right. 2010, New York, N.Y., London: Metropolitan Books, Profile Books.
3. Birkmeyer, J.D., Strategies for improving surgical quality--checklists and beyond. *N Engl J Med* 2010;363(20):1963-5.
4. Hearns, S., Checklists in emergency medicine. *Emergency Medicine Journal* 2018;35(9):530-531.
5. Goldhaber-Fiebert, S.N., S. Bereknyei Merrell, A.V. Agarwala, et al., Clinical Uses and Impacts of Emergency Manuals During Perioperative Crises. *Anesth Analg* 2020;131(6):1815-1826.
6. Burian, B.K., A. Clebone, K. Dismukes, et al., More Than a Tick Box: Medical Checklist Development, Design, and Use. *Anesth Analg* 2018;126(1):223-232.
7. Clebone, A., B.K. Burian, and A. Tung, Matching design to use: a task analysis comparison of three cognitive aid designs used during simulated crisis management. *Can J Anaesth* 2019;66(6):658-671.
8. Verdaasdonk, E.G., L.P. Stassen, P.P. Widhiasmara, et al., Requirements for the design and implementation of checklists for surgical processes. *Surg Endosc* 2009;23(4):715-26.

For peer review only - http://bmjopen.bmj.com/site/about/guidelines.xhtml

9. Haynes, A.B., T.G. Weiser, W.R. Berry, et al., A surgical safety checklist to reduce morbidity and mortality in a global population. *N Engl J Med* 2009;360(5):491-9.
10. de Vries, E.N., H.A. Prins, R.M. Crolla, et al., Effect of a comprehensive surgical safety system on patient outcomes. *N Engl J Med* 2010;363(20):1928-37.
11. Sotto, K.T., B.K. Burian, and M. Brindle, Impact of the WHO Surgical Safety Checklist Relative to Its Design and Intended Use: A Systematic Review and Meta-Meta-Analysis. *J Am Coll Surg* 2021.
12. Pronovost, P., D. Needham, S. Berenholtz, et al., An intervention to decrease catheter-related bloodstream infections in the ICU. *N Engl J Med* 2006;355(26):2725-32.
13. Pronovost, P.J., C.A. Goeschel, E. Colantuoni, et al., Sustaining reductions in catheter related bloodstream infections in Michigan intensive care units: observational study. *BMJ* 2010;340:c309.
14. Bion, J., A. Richardson, P. Hibbert, et al., 'Matching Michigan': a 2-year stepped interventional programme to minimise central venous catheter-blood stream infections in intensive care units in England. *BMJ Qual Saf* 2013;22(2):110-23.
15. LeBlanc, V.R., The effects of acute stress on performance: implications for health professions education. *Acad Med* 2009;84(10 Suppl):S25-33.
16. Dismukes, R., T.E. Goldsmith, and J.A. Kochan, *Effects of acute stress on aircrew performance: literature review and analysis of operational aspects (NASA/TM-2015-218930)*. 2015, National Aeronautics and Space Administration: Moffett Field, CA.
17. Arriaga, A.F., A.M. Bader, J.M. Wong, et al., Simulation-based trial of surgical-crisis checklists. *N Engl J Med* 2013;368(3):246-53.
18. Just, K.S., S. Hubrich, D. Schmidtke, et al., The effectiveness of an intensive care quick reference checklist manual--a randomized simulation-based trial. *J Crit Care* 2015;30(2):255-60.
19. Koers, L., M. van Haperen, C.G.F. Meijer, et al., Effect of Cognitive Aids on Adherence to Best Practice in the Treatment of Deteriorating Surgical Patients: A Randomized Clinical Trial in a Simulation Setting. *JAMA Surg* 2020;155(1):e194704.
20. Hall, C., D. Robertson, M. Rolfe, et al., Do cognitive aids reduce error rates in resuscitation team performance? Trial of emergency medicine protocols in simulation training (TEMPIST) in Australia. *Hum Resour Health* 2020;18(1):1.
21. Knoche, B.B., C. Busche, M. Grodd, et al., A simulation-based pilot study of crisis checklists in the emergency department. *Intern Emerg Med* 2021.
22. Dryver, E., J. Lundager Forberg, C. Hård Af Segerstad, et al., Medical crisis checklists in the emergency department: a simulation-based multi-institutional randomised controlled trial. *BMJ Qual Saf* 2021;30(9):697-705.
23. Simmons, W.R. and J. Huang, Operating Room Emergency Manuals Improve Patient Safety: A Systemic Review. *Cureus* 2019;11(6):e4888.
24. Ranganathan, P., J.H. Phillips, A.F. Attaallah, et al., The use of cognitive aid checklist leading to successful treatment of malignant hyperthermia in an infant undergoing cranioplasty. *Anesth Analg* 2014;118(6):1387.
25. Bereknyei Merrell, S., D.M. Gaba, A.V. Agarwala, et al., Use of an Emergency Manual During an Intraoperative Cardiac Arrest by an Interprofessional Team: A Positive-Exemplar Case Study of a New Patient Safety Tool. *Jt Comm J Qual Patient Saf* 2018;44(8):477-484.
26. Huang, J., F. Jiang, J.-F. Zhang, et al., The use of emergency manuals leading to successful treatment of severe bronchospasm. *The Journal of Medical Practice Management: MPM* 2018;33(5):330-332.

For peer review only - http://bmjopen.bmj.com/site/about/guidelines.xhtml

27. Goldhaber-Fiebert, S.N., A. Frackman, A.V. Agarwala, et al., Emergency manual peri-crisis use six years following implementation: Sustainment of an intervention for rare crises. *Journal of Clinical Anesthesia* 2023;87:111111.
28. Carayon, P., T.B. Wetterneck, A.J. Rivera-Rodriguez, et al., Human factors systems approach to healthcare quality and patient safety. *Appl Ergon* 2014;45(1):14-25.
29. Luedi, M.M. and M.B. Weinger, Emergency Manuals in Context: One Component of Resilient Performance. *Anesthesia & Analgesia* 2020;131(6):1812-1814.
30. Kapadia, M.R. and S.J. Ivatury, Transitioning Cognitive Aids for Training to Tools for Rescue in Clinical Practice. *JAMA Surgery* 2020;155(1):e194705-e194705.
31. Goldhaber-Fiebert, S.N. and C. Macrae, Emergency Manuals: How Quality Improvement and Implementation Science Can Enable Better Perioperative Management During Crises. *Anesthesiol Clin* 2018;36(1):45-62.
32. Burden, A.R., Z.J. Carr, G.W. Staman, et al., Does every code need a "reader?" improvement of rare event management with a cognitive aid "reader" during a simulated emergency: a pilot study. *Simul Healthc* 2012;7(1):1-9.
33. World Health Organization & WHO Patient Safety, *Implementation Manual - WHO Surgical Safety Checklist (First Edition)*. 2008, World Health Organization: https://apps.who.int/iris/handle/10665/70046.
34. Härtel, C., G. Prosen, R. Brown, et al., *European Core Curriculum for Emergency Medicine. Version 2.0*. 2019 [cited 27 july 2019], European Society for Emergency Medicine: https://eusem.org/images/Curriculum_2.0_WEB.pdf.
35. Civil Aviation Authority (UK) *CAP 708: Guidance on the Design, Presentation and Use of Electronic Checklists*. 2005.
36. Civil Aviation Authority (UK) *CAP 676: Guidance on the Design, Presentation and Use of Emergency and Abnormal Checklists*. 2006.
37. Burian, B.K., Design Guidance for Emergency and Abnormal Checklists in Aviation. *Proceedings of the Human Factors and Ergonomics Society Annual Meeting* 2006;50(1):106-110.
38. Hales, B., M. Terblanche, R. Fowler, et al., Development of medical checklists for improved quality of patient care. *Int J Qual Health Care* 2008;20(1):22-30.
39. Hepner, D.L., A.F. Arriaga, J.B. Cooper, et al., Operating Room Crisis Checklists and Emergency Manuals. *Anesthesiology* 2017;127(2):384-392.
40. Clebone, A., B.K. Burian, and A. Tung, The effect of cognitive aid design on the perceived usability of critical event cognitive aids. *Acta Anaesthesiol Scand* 2020;64(3):378-384.
41. Dryver, E., Emergency Medicine Checklist Compendium. 2022: Media-Tryck, Lund University.
42. Borshoff, D. and S. Weingart, The Resuscitation Crisis Manual. 2018: Leeuwin Press.
43. Goldhaber-Fiebert, S.N., N. Austin, E. Sultan, et al., *Emergency Manual: Cognitive aids for perioperative crises*. 2021: http://emergencymanual.stanford.edu
44. Robertson, D., *Emergency Protocols*, C. Hall, J. Mackenzie, and L. Murray, Editors. 2021: http://emergencyprotocols.org.au.
45. Borshoff, D., The limitations of crisis checklists. *Anesth Analg* 2014;118(6):1387-8.
46. Sollid, S.J.M. and A. Kämäräinen, The checklist, your friend or foe? *Acta Anaesthesiol Scand* 2020;64(1):4-5.

For peer review only - http://bmjopen.bmj.com/site/about/guidelines.xhtml

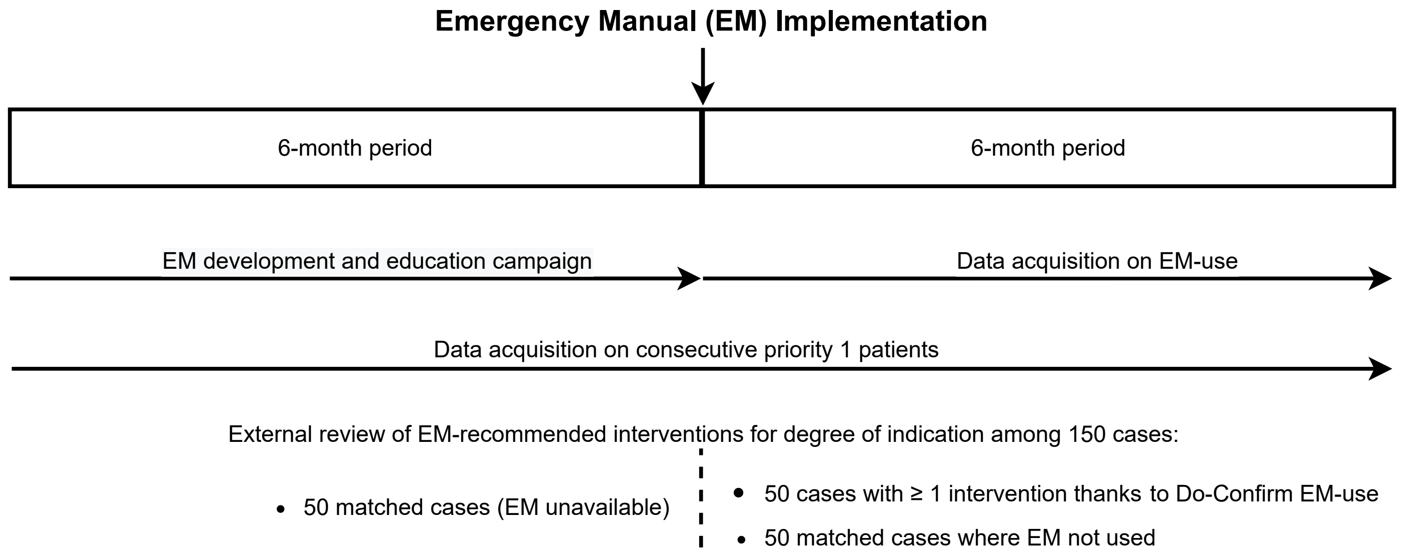
Emergency Manual (EM) Implementation
6-month period
6-month period
EM development and education campaign
Data acquisition on EM-use
Data acquisition on consecutive priority 1 patients
External review of EM-recommended interventions for degree of indication among 150 cases:
50 matched cases (EM unavailable)
50 cases with ≥ 1 intervention thanks to Do-Confirm EM-use
50 matched cases where EM not used

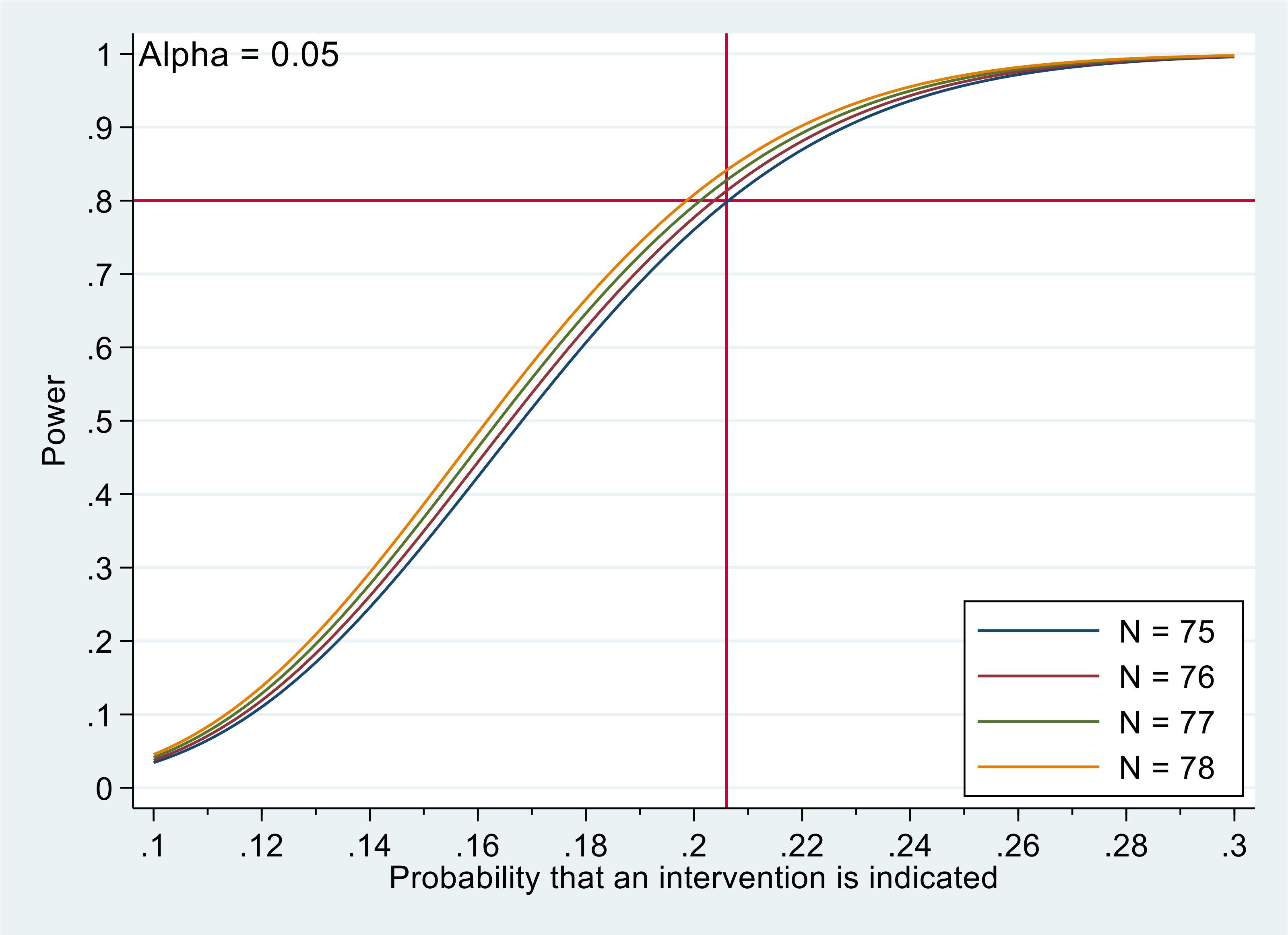
Alpha = 0.05
Power
Probability that an intervention is indicated
1
.9
.8
.7
.6
.5
.4
.3
.2
.1
0
.1
.12
.14
.16
.18
.2
.22
.24
.26
.28
.3
N = 75
N = 76
N = 77
N = 78

Appendix A: Structured Interview Guide for Reported Specific Cases

Introduction: You have requested to speak with us about the use of the Emergency Manual during the management of a specific patient in the resuscitation room. We are interested in understanding how the Emergency Manual is used, how it impacts on actual clinical care and how it can be improved.

Important information:
This project has been approved by the Swedish Ethical Review Authority
The names of specific health care personnel are not recorded.
This information obtained through this interview will not be used for any institutional process for assessing clinical management.

Background Information

Date of the interview:	
Date specific case managed:	
Interviewee's profession:	☐ Nursing assistant ☐ Nurse ☐ Resident ☐ Specialist. Physician's specialty:
Physician in charge of case	☐ Resident ☐ Specialist. Specialty:
Patient age and sex	____________ years. ☐ Male ☐ Female
Presenting complaint	
Suspected diagnosis	

Events or concerns relating to EM use (free text summary, professions of individuals recorded but names NOT recorded)

Emergency Manual Use

Sections of EM relevant to the event	
Mode of EM use	☐ Do-Confirm ☐ Read-Do ☐ Sampling

For peer review only

Assessed impact of EM use on patient care (clinical decisions regarding diagnosis, treatment; free text summary)

Summary of Impact of EM use on patient care: ☐ Negative ☐ Positive

Assessed impact of EM use on team members/teamwork (organization, communication; free text summary)

Summary of Impact of EM use on team members/teamwork: ☐ Negative ☐ Positive

Verbatim summary of how the impact of EM access on the specific case:

Suggestions for improvement (free text summary):

For peer review only

Reporting checklist for protocol of a clinical trial.

Based on the SPIRIT guidelines.

Instructions to authors

Complete this checklist by entering the page numbers from your manuscript where readers will find each of the items listed below.

Your article may not currently address all the items on the checklist. Please modify your text to include the missing information. If you are certain that an item does not apply, please write "n/a" and provide a short explanation.

Upload your completed checklist as an extra file when you submit to a journal.

In your methods section, say that you used the SPIRITreporting guidelines, and cite them as:

Chan A-W, Tetzlaff JM, Gøtzsche PC, Altman DG, Mann H, Berlin J, Dickersin K, Hróbjartsson A, Schulz KF, Parulekar WR, Krleža-Jerić K, Laupacis A, Moher D. SPIRIT 2013 Explanation and Elaboration: Guidance for protocols of clinical trials. BMJ. 2013;346:e7586

		Reporting Item	Page Number
**Administrative information**			
Title	#1	Descriptive title identifying the study design, population, interventions, and, if applicable, trial acronym	1
Trial registration	#2a	Trial identifier and registry name. If not yet registered, name of intended registry	3
Trial registration: data set	#2b	All items from the World Health Organization Trial Registration Data Set	n/a (used registry: clinicaltrials.gov)
Protocol version	#3	Date and version identifier	n/a (version 1)
Funding	#4	Sources and types of financial, material, and other support	20
Roles and responsibilities: contributorship	#5a	Names, affiliations, and roles of protocol contributors	1

For peer review only

Roles and responsibilities: sponsor contact information	#5b	Name and contact information for the trial sponsor	1
Roles and responsibilities: sponsor and funder	#5c	Role of study sponsor and funders, if any, in study design; collection, management, analysis, and interpretation of data; writing of the report; and the decision to submit the report for publication, including whether they will have ultimate authority over any of these activities	20
Roles and responsibilities: committees	#5d	Composition, roles, and responsibilities of the coordinating centre, steering committee, endpoint adjudication committee, data management team, and other individuals or groups overseeing the trial, if applicable (see Item 21a for data monitoring committee)	n/a
**Introduction**			
Background and rationale	#6a	Description of research question and justification for undertaking the trial, including summary of relevant studies (published and unpublished) examining benefits and harms for each intervention	5
Background and rationale: choice of comparators	#6b	Explanation for choice of comparators	n/a (the comparison is management with vs without access to an EM)
Objectives	#7	Specific objectives or hypotheses	8
Trial design	#8	Description of trial design including type of trial (eg, parallel group, crossover, factorial, single group), allocation ratio, and framework (eg, superiority, equivalence, non-inferiority, exploratory)	9
**Methods: Participants,**			

For peer review only

**interventions, and outcomes**			
Study setting	#9	Description of study settings (eg, community clinic, academic hospital) and list of countries where data will be collected. Reference to where list of study sites can be obtained	9
Eligibility criteria	#10	Inclusion and exclusion criteria for participants. If applicable, eligibility criteria for study centres and individuals who will perform the interventions (eg, surgeons, psychotherapists)	9
Interventions: description	#11a	Interventions for each group with sufficient detail to allow replication, including how and when they will be administered	10
Interventions: modifications	#11b	Criteria for discontinuing or modifying allocated interventions for a given trial participant (eg, drug dose change in response to harms, participant request, or improving / worsening disease)	15
Interventions: adherance	#11c	Strategies to improve adherence to intervention protocols, and any procedures for monitoring adherence (eg, drug tablet return; laboratory tests)	11
Interventions: concomitant care	#11d	Relevant concomitant care and interventions that are permitted or prohibited during the trial	11
Outcomes	#12	Primary, secondary, and other outcomes, including the specific measurement variable (eg, systolic blood pressure), analysis metric (eg, change from baseline, final value, time to event), method of aggregation (eg, median, proportion), and time point for each outcome. Explanation of the clinical relevance of chosen efficacy and harm outcomes is strongly recommended	13

For peer review only

Participant timeline	#13	Time schedule of enrolment, interventions (including any run-ins and washouts), assessments, and visits for participants. A schematic diagram is highly recommended (see Figure)	9 + Figure 1
Sample size	#14	Estimated number of participants needed to achieve study objectives and how it was determined, including clinical and statistical assumptions supporting any sample size calculations	14 + Figure 2
Recruitment	#15	Strategies for achieving adequate participant enrolment to reach target sample size	n/a (consecutive priority 1 patients treated in the ED)
**Methods: Assignment of interventions (for controlled trials)**			
Allocation: sequence generation	#16a	Method of generating the allocation sequence (eg, computer-generated random numbers), and list of any factors for stratification. To reduce predictability of a random sequence, details of any planned restriction (eg, blocking) should be provided in a separate document that is unavailable to those who enrol participants or assign interventions	n/a (one arm study)
Allocation concealment mechanism	#16b	Mechanism of implementing the allocation sequence (eg, central telephone; sequentially numbered, opaque, sealed envelopes), describing any steps to conceal the sequence until interventions are assigned	n/a
Allocation: implementation	#16c	Who will generate the allocation sequence, who will enrol participants, and who will assign participants to interventions	n/a

For peer review only

Blinding (masking)	#17a	Who will be blinded after assignment to interventions (eg, trial participants, care providers, outcome assessors, data analysts), and how	14
Blinding (masking): emergency unblinding	#17b	If blinded, circumstances under which unblinding is permissible, and procedure for revealing a participant's allocated intervention during the trial	n/a (no blinding for intervention, i.e. access to checklists)
**Methods: Data collection, management, and analysis**			
Data collection plan	#18a	Plans for assessment and collection of outcome, baseline, and other trial data, including any related processes to promote data quality (eg, duplicate measurements, training of assessors) and a description of study instruments (eg, questionnaires, laboratory tests) along with their reliability and validity, if known. Reference to where data collection forms can be found, if not in the protocol	12
Data collection plan: retention	#18b	Plans to promote participant retention and complete follow-up, including list of any outcome data to be collected for participants who discontinue or deviate from intervention protocols	n/a (consecutive patients management in the ED)
Data management	#19	Plans for data entry, coding, security, and storage, including any related processes to promote data quality (eg, double data entry; range checks for data values). Reference to where details of data management procedures can be found, if not in the protocol	12
Statistics: outcomes	#20a	Statistical methods for analysing primary and secondary outcomes. Reference to	16

For peer review only

		where other details of the statistical analysis plan can be found, if not in the protocol	
Statistics: additional analyses	#20b	Methods for any additional analyses (eg, subgroup and adjusted analyses)	16
Statistics: analysis population and missing data	#20c	Definition of analysis population relating to protocol non-adherence (eg, as randomised analysis), and any statistical methods to handle missing data (eg, multiple imputation)	n/a
**Methods: Monitoring**			
Data monitoring: formal committee	#21a	Composition of data monitoring committee (DMC); summary of its role and reporting structure; statement of whether it is independent from the sponsor and competing interests; and reference to where further details about its charter can be found, if not in the protocol. Alternatively, an explanation of why a DMC is not needed	n/a (checklist use is not mandatory; there is no evidence that checklist access is hazardous to patient management; teams can request immediate contact with the investigators to report specific events associated with checklist use)
Data monitoring: interim analysis	#21b	Description of any interim analyses and stopping guidelines, including who will have access to these interim results and make the final decision to terminate the trial	n/a
Harms	#22	Plans for collecting, assessing, reporting, and managing solicited and spontaneously reported adverse events and other unintended effects of trial interventions or trial conduct	15
Auditing	#23	Frequency and procedures for auditing trial conduct, if any, and whether the process will be independent from investigators and the sponsor	n/a (this study focuses on how teams use a new tool)
**Ethics and dissemination**			
Research ethics	#24	Plans for seeking research ethics committee	18

For peer review only

approval		/ institutional review board (REC / IRB) approval	
Protocol amendments	#25	Plans for communicating important protocol modifications (eg, changes to eligibility criteria, outcomes, analyses) to relevant parties (eg, investigators, REC / IRBs, trial participants, trial registries, journals, regulators)	n/a (consecutive unselected patients managed in the emergency room)
Consent or assent	#26a	Who will obtain informed consent or assent from potential trial participants or authorised surrogates, and how (see Item 32)	17
Consent or assent: ancillary studies	#26b	Additional consent provisions for collection and use of participant data and biological specimens in ancillary studies, if applicable	n/a (no such data)
Confidentiality	#27	How personal information about potential and enrolled participants will be collected, shared, and maintained in order to protect confidentiality before, during, and after the trial	12
Declaration of interests	#28	Financial and other competing interests for principal investigators for the overall trial and each study site	20
Data access	#29	Statement of who will have access to the final trial dataset, and disclosure of contractual agreements that limit such access for investigators	13
Ancillary and post trial care	#30	Provisions, if any, for ancillary and post-trial care, and for compensation to those who suffer harm from trial participation	n/a
Dissemination policy: trial results	#31a	Plans for investigators and sponsor to communicate trial results to participants, healthcare professionals, the public, and other relevant groups (eg, via publication, reporting in results databases, or other data	18

For peer review only

		sharing arrangements), including any publication restrictions	
Dissemination policy: authorship	#31b	Authorship eligibility guidelines and any intended use of professional writers	n/a
Dissemination policy: reproducible research	#31c	Plans, if any, for granting public access to the full protocol, participant-level dataset, and statistical code	n/a
**Appendices**			
Informed consent materials	#32	Model consent form and other related documentation given to participants and authorised surrogates	n/a (see above)
Biological specimens	#33	Plans for collection, laboratory evaluation, and storage of biological specimens for genetic or molecular analysis in the current trial and for future use in ancillary studies, if applicable	n/a (no such data collected)

None The SPIRIT Explanation and Elaboration paper is distributed under the terms of the Creative Commons Attribution License CC-BY-NC. This checklist can be completed online using https://www.goodreports.org/, a tool made by the EQUATOR Network in collaboration with Penelope.ai

For peer review only
